# Small Molecule Antagonists of NAADP-Induced Ca^2+^ Release in T-Lymphocytes Suggest Potential Therapeutic Agents for Autoimmune Disease

**DOI:** 10.1038/s41598-018-34917-3

**Published:** 2018-11-13

**Authors:** Bo Zhang, Joanna M Watt, Chiara Cordiglieri, Werner Dammermann, Mary F. Mahon, Alexander Flügel, Andreas H. Guse, Barry V. L. Potter

**Affiliations:** 1 Medicinal Chemistry & Drug Discovery, Department of Pharmacology, University of Oxford, Mansfield Road, Oxford, OX1 3QT UK; 2 Wolfson Laboratory of Medicinal Chemistry, University of Bath, Dept. of Pharmacy and Pharmacology, Claverton Down, Bath, BA2 7AY UK; 3 Max-Planck-Institute for Neurobiology, Martinsried, Germany; 4 The Calcium Signalling Group, Department of Biochemistry and Molecular Cell Biology, University Medical Centre Hamburg-Eppendorf, Martinistrasse 52, D-20246 Hamburg, Germany; 5 Department of Chemistry, University of Bath, Claverton Down, Bath, BA2 7AY UK; 6 University Medical Center Göttingen, Institute for Multiple Sclerosis Research, Department of Neuroimmunology, Von-Siebold-Str. 3a, 37075 Göttingen, Germany; 7 Present Address: Imaging Facility, National Institute for Molecular Genetics (INGM), v. F. Sforza, 35-20122 Milan, Italy; 8 Present Address: Brandenburg Medical School, University Hospital Brandenburg, Center of Internal Medicine II, Hochstraße 29, 14770 Brandenburg an der Havel, Germany

## Abstract

Nicotinic acid adenine dinucleotide phosphate (NAADP) is the most potent Ca^2+^-releasing second messenger known to date, but the precise NAADP/Ca^2+^ signalling mechanisms are still controversial. We report the synthesis of small-molecule inhibitors of NAADP-induced Ca^2+^ release based upon the nicotinic acid motif. Alkylation of nicotinic acid with a series of bromoacetamides generated a diverse compound library. However, many members were only weakly active or had poor physicochemical properties. Structural optimisation produced the best inhibitors that interact specifically with the NAADP/Ca^2+^ release mechanism, having no effect on Ca^2+^ mobilized by the other well-known second messengers d-*myo*-inositol 1,4,5-trisphosphate [Ins(1,4,5)P_3_] or cyclic adenosine 5′-diphospho-ribose (cADPR). Lead compound **(2)** was an efficient antagonist of NAADP-evoked Ca^2+^ release *in vitro* in intact T lymphocytes and ameliorated clinical disease *in vivo* in a rat experimental autoimmune encephalomyelitis (EAE) model of multiple sclerosis. Compound **(3)** (also known as BZ194) was synthesized as its bromide salt, confirmed by crystallography, and was more membrane permeant than **2**. The corresponding zwitterion (**3a**), was also prepared and studied by crystallography, but **3** had more desirable physicochemical properties. **3** Is potent *in vitro* and *in vivo* and has found widespread use as a tool to modulate NAADP effects in autoimmunity and cardiovascular applications. Taken together, data suggest that the NAADP/Ca^2+^ signalling mechanism may serve as a potential target for T cell- or cardiomyocyte-related diseases such as multiple sclerosis or arrhythmia. Further modification of these lead compounds may potentially result in drug candidates of clinical use.

## Introduction

Ca^2+^ is one of the major signal transduction pathways in living cells and is involved in the regulation of many important cellular processes from proliferation to apoptosis^1,2^. In both invertebrate and mammalian systems the release of intracellular Ca^2+^ is mediated through three second messengers: D-*myo*-inositol-1,4,5-triphosphate (Ins(1,4,5)P_3_), cyclic adenosine 5′-diphosphoribose (cADPR) and nicotinic acid adenine dinucleotide 2′-phosphate (NAADP). NAADP was first discovered and identified as a Ca^2+^-mobilizing second messenger in sea urchin egg homogenate (SUH)^3–5^. It is structurally related to nicotinamide adenine dinucleotide phosphate (NADP) that has an amide on the pyridinium ring instead of a carboxylic acid. Remarkably, this single functional group interconversion confers total specificity (as NADP is completely inactive in Ca^2+^ mobilisation) and makes NAADP the most potent Ca^2+^-mobilizing second messenger known to date; typically low nanomolar concentrations trigger the release of Ca^2+^ from intracellular stores in many different cells^6,7^. NAADP activates intracellular Ca^2+^ channels whose exact location and molecular identity is still under debate^8–10^. Elucidation of the NAADP/Ca^2+^ signalling mechanism and purification and characterization of the NAADP receptor may be aided by development of stable, membrane-permeant, chemical biology tools that specifically activate or antagonize this pathway.

NAADP, and its signalling mechanisms may serve as targets for the development of clinical agents for Ca^2+^-related diseases, such as multiple sclerosis (MS)^11^. MS and other autoimmune diseases are caused and controlled by autoreactive T cells. Activation of T cells initiates invasion into the central nervous system (CNS)^12^. This activates multiple intramolecular pathways, including elevation of the free cytosolic Ca^2+^ concentration that is essential for proliferation. NAADP may act as a trigger for Ca^2+^ signalling in T cells and a functional NAADP/Ca^2+^ mechanism is essential for T-cell Ca^2+^ signalling^7^. There are currently no curative therapies approved for MS^13,14^. Specific inhibitors of NAADP-induced Ca^2+^-release, may interfere with T cell activation and proliferation and help to elucidate the mechanism behind diseases such as MS.

NAADP and its derivatives were first synthesized enzymatically from NADP and analogues using a base-exchange reaction mediated by ADP-ribosyl cyclase^15^. Some of us reported previously a flexible route to NAADP via total chemical synthesis of NADP^16,17^. However, the cost and practicability of the synthesis may limit the production of further analogues. Furthermore, both routes rely on a base-exchange process in the last step, limiting potential NAADP analogues to NADP-based substrates accepted by ADP-ribosyl cyclase. NAADP analogues generated *via* these routes have generated an outline structure-activity relationship^18^. Labelled [^32^P]NAADP has been synthesized^19^ and was used as a radioactive tag in attempts to purify the NAADP-binding protein^20^. Other compounds such as triazine dyes^21^ have been tried in the affinity purification of NAADP receptor. Such dyes, however, are not membrane permeant, not structurally related to NAADP and also lack selectivity as they interact with Ins(1,4,5)P_3_ receptors. A selective cell-permeant NAADP antagonist Ned-19 was discovered by virtual screening^22^ and has found widespread use in biological studies^23,24^. Ned-19 has a 3D-shape and electrostatics that are similar to NAADP, but their chemical (2D) structures are unrelated. The plant two-pore channel (TPC) from *Arabidopsis thaliana* was recently crystallized with Ned-19 allosterically bound^25^.

NAADP possesses pyrophosphate, phosphate groups and a carboxylic acid group that are negatively charged at physiological pH that pose challenges for synthetic design and membrane permeability. Cell-permeant acetoxymethyl protected NAADP (NAADP-AM) was synthesized^26^ and has enabled the study of the NAADP/Ca^2+^ mechanism in intact cells. The AM groups are in principle cleaved inside the cell by the action of cytosolic esterases. However, this ligand, prepared by poorly characterised pan-derivatisation of both nucleotide motifs and the pyrophosphate, is unsurprisingly fraught with serious stability and homogeneity issues and is very unsatisfactory from a structural and chemical perspective. Two caged NAADP analogues bearing a 1-(2-nitrophenyl)ethanol (NPE)^27^ and a 1-[(2-nitro-4,5-dimethoxy)phenyl]ethanol (DMNPE)^28^ group were synthesized which allowed NAADP to be administered in a controlled fashion by release of NAADP upon UV irradiation.

Despite this progress such derivatives are generally unstable. The cell permeant analogues are even less stable due to the reactivity of the caging/protecting groups, as masking the negative charges to confer membrane permeability introduces instability towards nucleophiles and neighbouring groups. The difficult synthesis and purification of such NAADP analogues also severely restricts the number and scale of compounds that can realistically be made. Such NAADP derivatives are therefore not sufficient for further application and new stable small-molecule NAADP modulators are required^29^, most ideally those of markedly reduced structural complexity.

**Figure 1:**
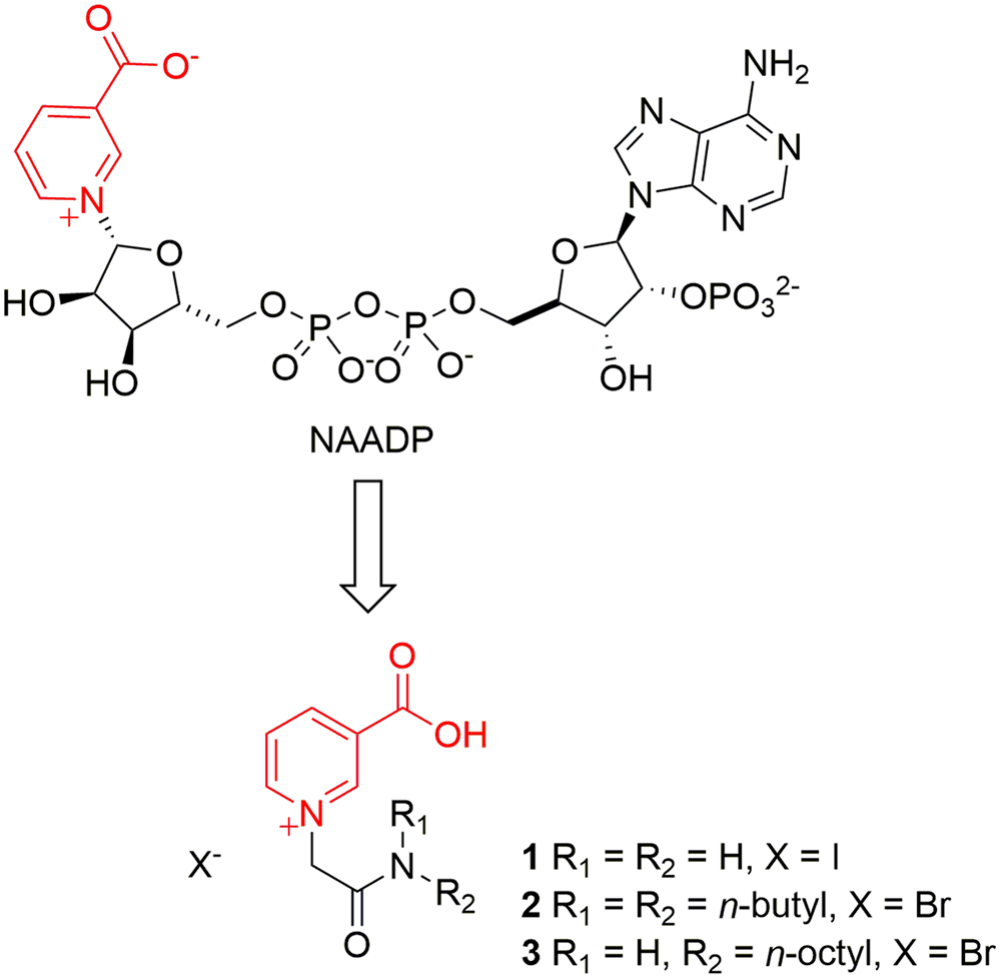
Design concept for small-molecule NAADP inhibitors.

An early SAR study of NAADP analogues revealed that both the pyridinium ring and the negative charge at the 3-position are crucial for Ca^2+^-mobilizing activity^15^. Replacement of the carboxylic acid by an amide group (as in NADP) or its removal resulted in complete loss of activity, indicating that the nicotinic acid moiety is essential for any NAADP-analogue activity^18^. A later study confirmed importance for both binding affinity and Ca^2+^ mobilizing activity^30^. Thus, rational design of small molecule NAADP analogues might initially focus on mimicking just the nicotinic acid part. Indeed, we previously showed that nicotinic acid alone, in principle, antagonized NAADP-evoked signalling^31^. Based on the above studies and with inspiration from the natural alkaloid trigonelline^32^, the prototype (**1**) was synthesized and pharmacologically evaluated (Fig. 1)^33^. The acetamide side chain was designed to be amenable to further modifications. Acetamide **1** inhibited Ca^2+^ release induced by NAADP in SUH, but not by cADPR or Ins(1,4,5)P_3_, indicating specificity for the NAADP/Ca^2+^ mechanism. **1** Also competed for binding with [^32^P]NAADP in a concentration-dependent manner^33^ and external administration abolished caged NAADP-induced Ca^2+^ release in sea urchin eggs, demonstrating cell permeability. Importantly, it could also antagonize NAADP-dependent Ca^2+^ oscillations induced by externally added cholecystokinin (CCK) in murine pancreatic acinar cells.

However, whilst preliminary data suggested that **1** works well in intact SUH and in mammalian pancreatic acinar cells^33^ it is difficult to handle and also appears to be inactive in some cells that utilize NAADP (e.g. in T cells). We now describe the synthesis and pharmacological evaluation of related lead analogues to probe the structure-activity relationship of **1** and to optimize physicochemical properties. Analogues were evaluated initially in T-lymphocytes and several active compounds discovered. Amongst these (**2**) and particularly (**3**) (Fig. 1), were extensively studied both *in vitro* and *in vivo;* data with compound **3** were published elsewhere^31^. Results indicate that such optimized analogues can be used as stable, membrane permeant tools to investigate NAADP/Ca^2+^ signalling. *In vivo* study of such compounds confirmed that the NAADP signalling pathway might be a potential novel target for development of agents for clinical applications in T-lymphocyte or cardiomyocyte related diseases.

## Results and Discussion

### Synthesis

**Figure 2:**
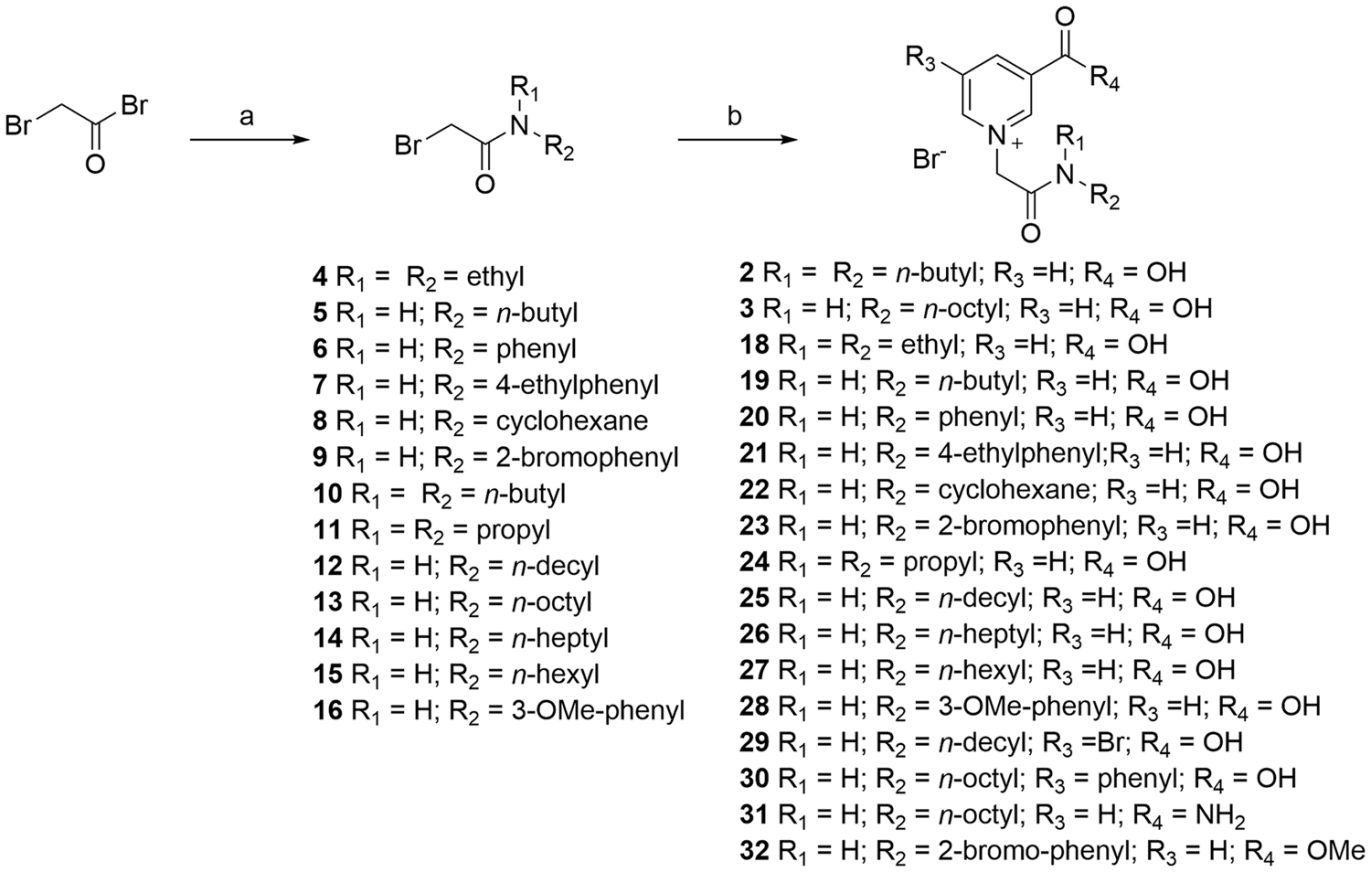
Synthesis of small-molecule NAADP analogues. Reagents and conditions*:* (**a**) R_1_R_2_NH, ether, ice-salt bath; (**b**) Nicotinic acid (or nicotinic acid derivative), DMF, 60–70 °C, 16 h.

Small-molecule NAADP analogues were prepared by alkylation of nicotinic acid derivatives with a series of bromoacetamides (Fig. 2). Bromoacetamides (**4–16**) were produced in good to excellent yield by treatment of bromoacetyl bromide with selected amines at low temperature. Treatment of the acetamides with nicotinic acid (or nicotinic acid derivatives), generated the desired pyridinium salts **2**, **3** and (**18–32**). Difficulties encountered during purification using column chromatography, because of the high polarity of both the carboxylic acid group and the positive charge on nitrogen, were overcome by precipitation using slow addition of ether to a methanol solution followed by several crystallizations from acetone/MeOH.

**Figure 3:**
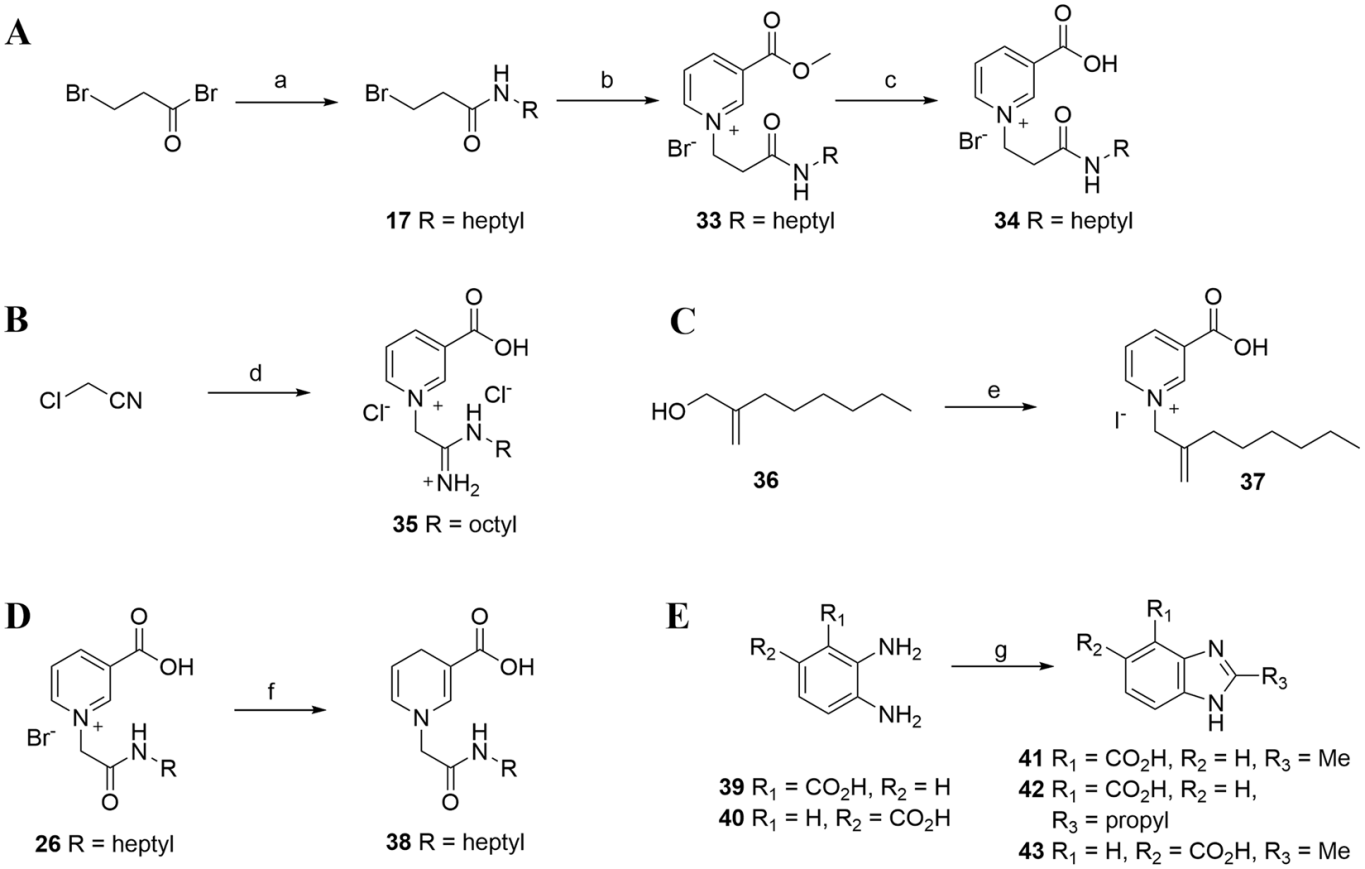
Synthesis of further small-molecule NAADP analogues. Reagents and conditions: (**A**) *n*-Heptylamine, ether, ice-salt bath; (**B**) nicotinic acid methyl ester, DMF, 60–70 °C, 16 h; (**C**) HBr (48%), 60 °C, 16 h; (**D**) (i) NaOMe, MeOH, octylamine hydrochloride, rt; (ii) nicotinic acid, DMF, 60–70 °C, 16 h; (**E**) (i) PPh_3_, I_2_, imidazole, rt; ii) nicotinic acid, DMF, 60–70 °C, 16 h; (**F**) Na_2_S_2_O_4_, NaHCO_3_, H_2_O/MeOH, rt; (**G**) 4 M HCl, reflux 1H^35^.

Other analogues bearing an alkene and an acetamidine group were also prepared to ascertain the importance of the amide motif linked to the nitrogen atom of nicotinic acid. Compound (**34**) which has a two-carbon chain between the nitrogen and the amide bond, was conveniently prepared under the usual conditions using bromopropionyl chloride rather than bromoacetyl bromide as a starting material (Fig. 3A). Acetamidine analogue (**35**) was synthesized from chloroacetronitrile that was first treated with NaOMe followed by *n*-octylamine hydrochloride to form the *N*-octyl substituted choloracetamidine^34,35^. This intermediate was reacted *in situ* with nicotinic acid to afford **35** in 22% yield (Fig. 3B). Alkene (**37)** was synthesized in two steps from commercially available *n*-octanol via a Mannich reaction^36^ followed by a NaBH_4_ reduction. The hydroxyl intermediate (**36**) thus obtained^37^ was then converted to its iodo-derivative and reacted with nicotinic acid to give (**37**) in 55% yield (Fig. 3C). As pyridinium salts can be reduced to the 1,4-dihydropyridine form using Na_2_S_2_O_4_/NaHCO_3_^38^, we designed compound (**38**) to act as a potential pro-drug of the pyridinium compound **26** (Fig. 3D). We hoped that removing the positively charged nitrogen in the pyridinium ring would generate a more cell-permeant analogue that in principle could be oxidized back to the active form within cells.

The amide may increase the rigidity of the analogue, and we therefore also synthesized compounds (**41–43**) based on the report of White *et al*.^39^ (Fig. 3E), where such compounds have been used as inhibitors of the DNA repair enzyme poly(ADP-ribose) polymerase.

### *In vitro* studies and structure activity relationship (SAR)

**Figure 4:**
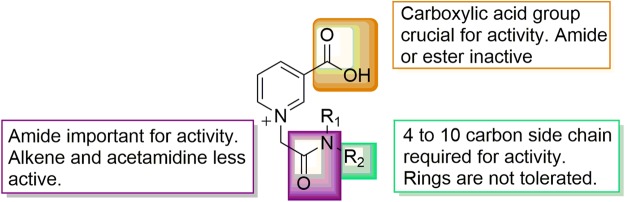
Outline structure-activity relationship.

**Figure 5:**
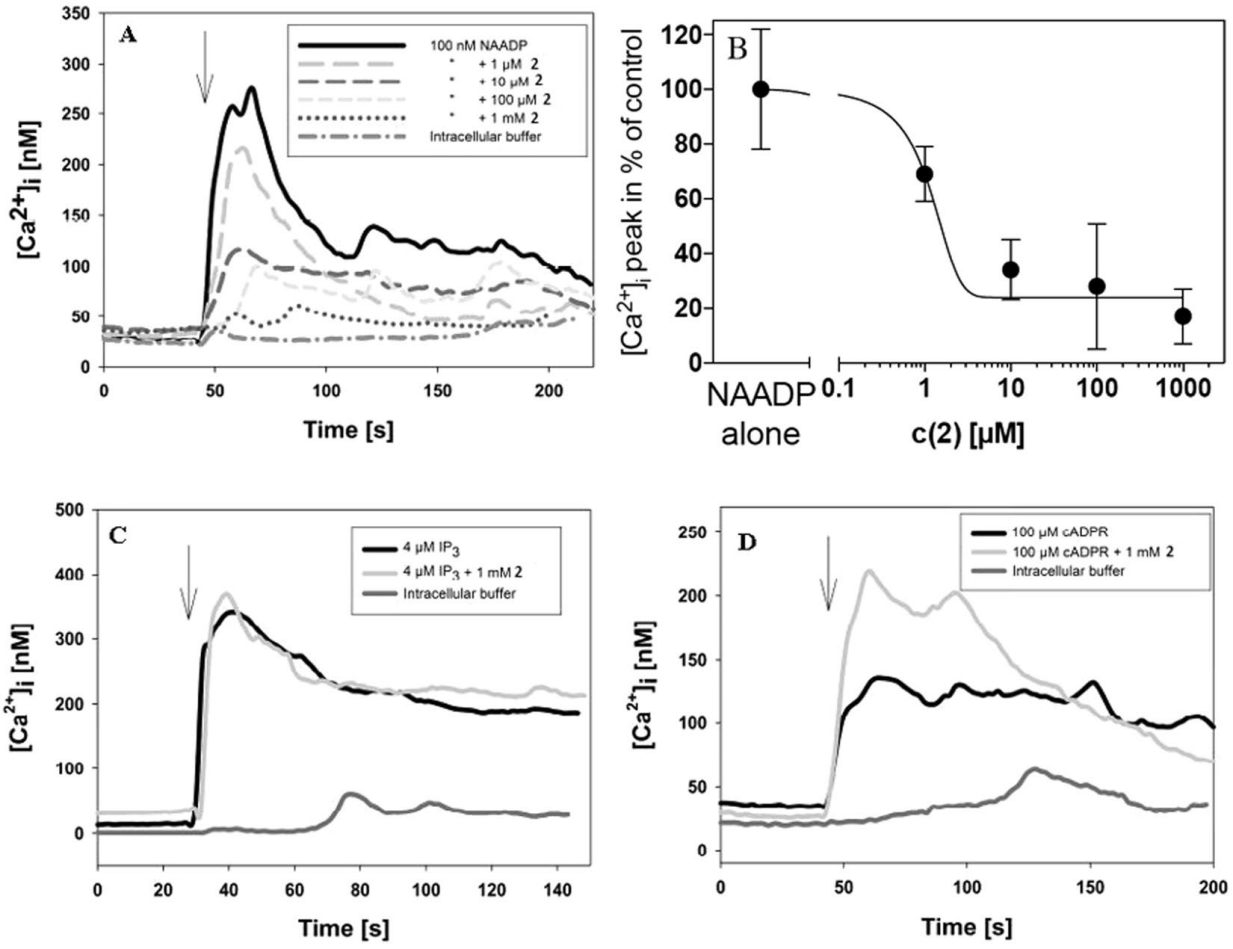
Effects of the NAADP antagonist **2** on Ca^2+^ signalling in single Jurkat T-lymphocytes. Jurkat T cells were loaded with Fura2/AM and calcium imaging and microinjection were carried out as previously described^50^. Arrows indicate time point of microinjection. (**A**), T cells were co-injected with 100 nM NAADP and increasing concentrations of **2** (n = 3–9). (**B**), Concentration-response curve showing the inhibitory effect of **2**. (**C**), Co-injection of 4 µM InsP_3_ and 1 mM **2** and of 4 µM InsP_3_ or buffer alone (n = 4–7). (**D**), Co-injection of 100 µM cADPR and 1 mM **2** and of 100 µM cADPR or buffer alone (n = 4–9). Data are mean ± s.e.m.

Novel NAADP analogues were evaluated in rat effector T cells specific for myelin-basic protein using [^3^H]deoxy-thymidine incorporation, as published previously^31^. Among different analogues **1–3, 21–23, 25** only **2** and **3** concentration-dependently antagonized antigen-dependent re-activation of rat effector T cells (Fig. 5 for the activity of **2**, and published data for **3**^31^). Although only a small library of NAADP analogues was synthesized, two inhibitors of antigen-dependent re-activation of rat effector T cells have been discovered that allow us to establish a very preliminary structure-activity relationship (SAR) for these small molecule antagonists (Fig. 4). The first conclusion relates to the C-3 position of the pyridinium ring: here, the carboxyclic acid moiety is crucial since the corresponding amide **31** or esters **32** are inactive. This might be expected, since NADP is inactive in Ca^2+^ release.

An alkyl side chain attached to the amide linker was introduced to improve cell permeability compared to **1**. Analogues with single alkyl chains; *n*-butyl **19**, *n*-hexyl **27**, *n*-heptyl **26**, *n*-octyl **3** and *n*-decyl **25**, were synthesized. It seems that apart from the nicotinic acid moiety, a side chain with greater than 4 but less than 10-carbon atoms is required for activity in intact T cells. Compounds with 10- or more carbon side-chains could not be studied quantitatively due to their poor solubility in biological buffers. Rings at the amide side-chain **21, 22, 23** are not tolerated. Hence an optimum balance between solubility and membrane permeability at this stage was achieved with eight carbons, spread over two chains **2** or one **3**. The amide and its position relative to the nicotinamide also appear crucial for activity; increasing the length of the carbon tether to the nicotinamide **34**, replacing the amide with an acetamidine **35** or alkene **37** all significantly decreased or abolished activity (data not shown).

### Further *in vitro* studies with lead compound **2**

Co-injection of **2** almost completely antagonized the effect of NAADP with an IC_50_ of 1.7 ± 0.8 µM; highest inhibition (approx. 80%) was obtained at 1 mM **2** (Fig. 5A,B). Evidence for the specificity of the inhibitory effect of **2** was obtained by co-injection with d-*myo*-Ins(1,4,5)P_3_ or cADPR. In these experiments d-*myo*-Ins(1,4,5)P_3_ induced a similar Ca^2+^ signal consisting of a rapid (peak) and sustained (plateau) phase as compared to NAADP. However, no difference between the presence and absence of **2** was observed (Fig. 5C). Similarly, Ca^2+^ signalling induced by microinjection of cADPR was not affected by **2** (Fig. 5D). Unexpectedly, the effect of cADPR was even enhanced in the presence of **2**; however, the underlying mechanism is unclear.

**Figure 6:**
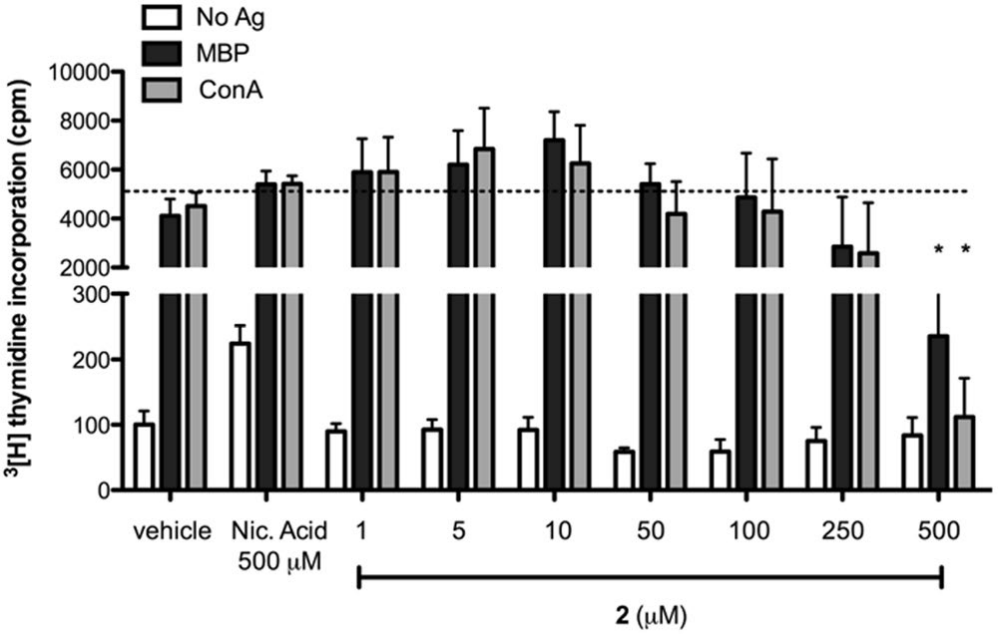
Inhibitory effect of **2** on antigen (MBP, black bars) and mitogen (ConA, grey bars) induced proliferation in T_MBP_ cells, as evaluated via [^3^H]-thymidine incorporation. White bars show basal proliferative levels of cells in absence of specific activation. Data are presented as mean ± SEM of five independent experiments. Vehicle and nicotinic acid (500 µM) were used as internal controls. Two-way ANOVA test shows statistically relevant differences in proliferation upon incubation with high doses of **2**, when compared to vehicle (threshold dotted line); (*)p < 0.05.

To determine the role of the NAADP/Ca^2+^ signalling system in T cell receptor/CD3 mediated activation, we studied the effect of **2** on stimulation of rat encephalitogenic CD4-positive^40^ T cells (T_MBP_ cells) *via* CD3 crosslinking. Here, **2** was shown to block both myelin-basic protein (MBP) and ConA-mediated proliferation (Fig. 6).

### *In vivo* studies

**Figure 7:**
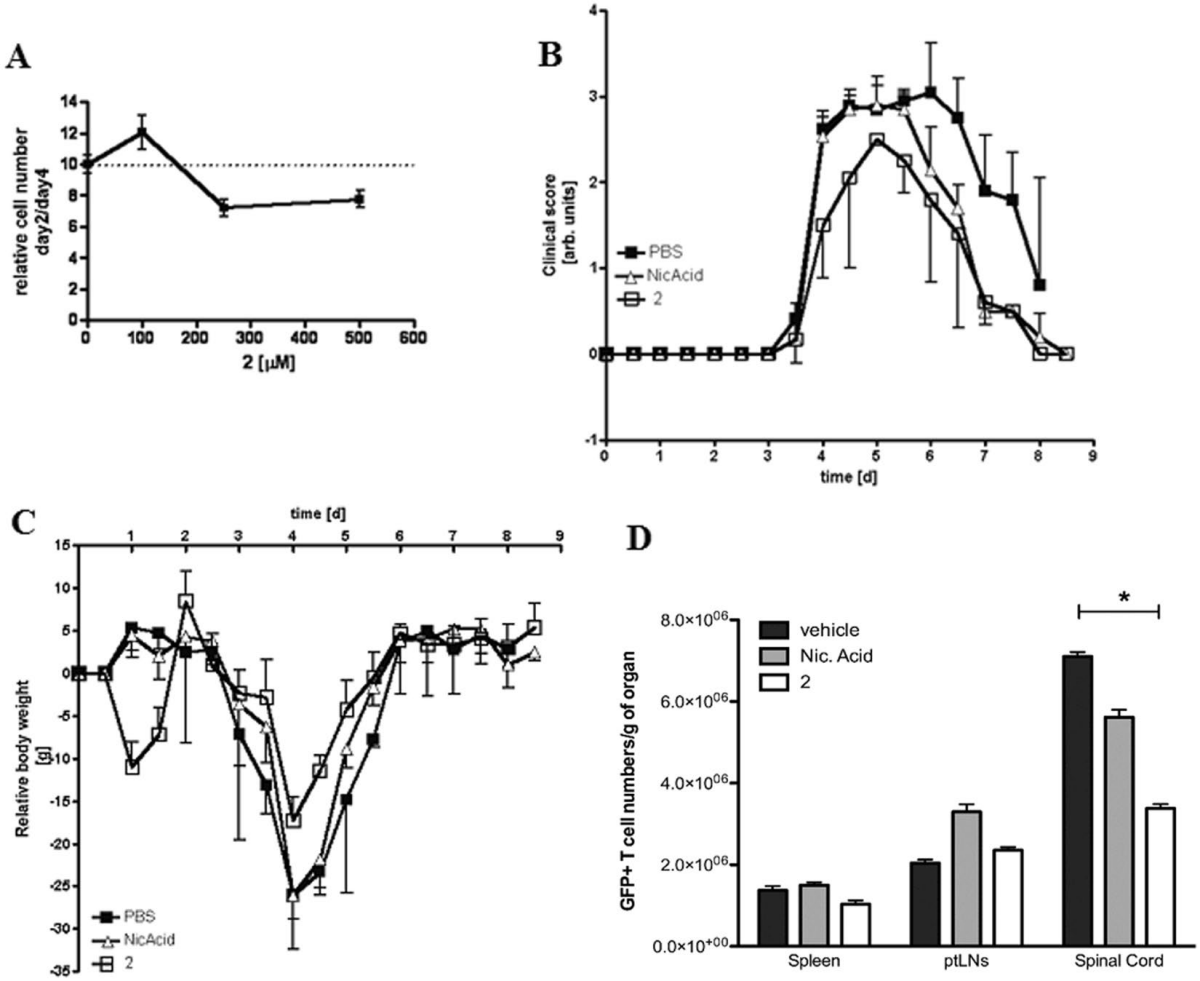
(**A**) Toxicity assay of **2** on non-proliferating myelin-basic protein (MBP) specific, CD4^+^ rat T cell blasts; (**B** and **C**) Protective effect of **2** in transfer experimental autoimmune encephalomyelitis (EAE); Animals (6 per group, body weight approx. 150 g) were injected i.p. twice per day with either PBS (vehicle control), nicotinic acid (50 μmol/100 g body weight), or **2** (50 μmol/100g body weight). Clinical scores indicate the degree of paralysis of tail and legs – as previously reported^42^. Data are presented as mean ± SD from one representative experiment (n = 6) of two independently conducted *in vivo* studies; (**D**) Effect of **2** on the localization of encephalitogenic T cells on day 4 post transfer in transfer experimental autoimmune encephalomyelitis (EAE); Animals (4 per group, body weight approx. 150 g) were injected i.p. twice per day with either PBS (vehicle control), nicotinic acid (50 μmol/100 g body weight), or **2** (50 μmol/100 g body weight). Animals were sacrificed on day 4 and the number of T_MBP-GFP_ cells was determined for the organs displayed (n = 4 per group). Data are corrected for organ mass and presented as mean ± SEM from two independent experiments. Non parametric Kruskal-Wallis test shows statistical relevant differences (p < 0.05) in spinal cord migrating T_MBP-GFP_ cells following treatment with **2**, as compared to vehicle-treated animals.

Increasing concentrations of **2** up to 500 μM did not significantly reduce the number of T_MBP_ cell blasts when incubated with these cells for 48 h, indicating that it does not have a large cytotoxic effect on resting cells, but specifically affects proliferating T cells (Fig. 7A). Therefore, **2** was studied *in vivo* in rat experimental autoimmune encephalomyelitis (EAE), a T cell-mediated animal model for MS^12^. Animals injected with PBS (vehicle) or nicotinic acid (as a control compound with some structural similarities to **2**) developed severe paralysis of tail and rear legs due to the inflammation. Clinical symptoms started on day 3.5 and reached their maxima on day 4 to 6.5. Between day 3.5 and day 6, no difference between PBS and nicotinic acid was observed. Animals treated with **2** showed a less rapid increase of symptoms, a decreased maximum score, a more rapid decline of clinical symptoms (*p* < 0.05 *vs*. either PBS or nicotinic acid, Mann-Whitney comparisons in EAE peak phase, day 4–8; Fig. 7B) and less decrease in body weight compared to the other two groups (*p* < 0.05 *vs*. either PBS or nicotinic acid, Mann-Whitney comparisons in EAE peak phase, day 3–5; Fig. 7C). Furthermore, the number of autoimmune T_MBP_ cells invading the CNS was reduced by 50%, upon treatment with 2 (*p* < 0.05 *vs*. either PBS or nicotinic acid, ANOVA), while the T cells in parathymic lymph nodes (LNs) and spleen were found to be slightly increased (Fig. 7D). It is known from previous studies^41^ that intravenously transferred encephalitogenic T cells first move to the spleen and para-thymic lymph nodes before reaching the CNS where they are re-activated by recognizing their target autoantigen (day 4 post transfer). This re-activation is essential for the recruitment of immune cells into the CNS and thus it is crucial for the induction of CNS inflammation and clinical disease. Since GFP-transduced encephalitogenic T cells were used for the transfer EAE experiment (T_MBP-GFP_ cells^40^), the numbers and localization of these cells could be determined on day 4 post transfer. For the animals treated with **2**, the number of CNS-infiltrating T_MBP-GFP_ cells decreased by about 50% (Fig. 7D). In addition, the number of T cells was reduced significantly. Our data thus strongly suggest that inhibition of NAADP/Ca^2+^ signalling will block the re-activation of effector T cells and render beneficial effects to EAE animals.

**Figure 8:**
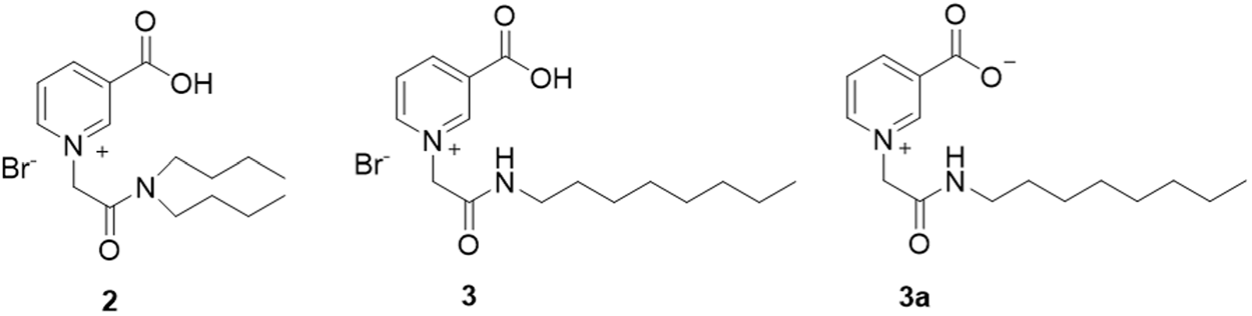
The structures of (**2**), the bromide salt (**3**) and the zwitterionic form (**3a**).

Further *in vitro* studies with different batches of **2** (Supplementary Fig. S4) suggested possible less than optimal membrane permeability of **2**, as some batch-dependent biological variability was noted in proliferation blockade efficacy, despite all batches of material showing equal inhibitory effects when co-injected with NAADP. Although **2** is uncharged overall as the zwitterion, the polar structure possibly limits its membrane permeability. We sought to optimize the structure of **2** and to improve cell permeability and this was achieved through modification of **2** to produce **3** (BZ194) (Fig. 8), representing structurally the rearrangement of the di-substituted-amide of **2** to the mono-substituted **3**; the hydrophobic tails in the former have the same number of carbon atoms as in **3**, but in **2** there are two tails (2 × 4 carbon atoms) and in **3** only one (1 × 8 carbon atoms). **3** Thus has the same molecular formula as **2**, but is mono *N*-substituted, and is less water-soluble. It has much better cell permeability than **2** and shows highly reproducible inhibitory effects in intact T cells^31^. Like **2**, **3** is an inhibitor of NAADP-induced Ca^2+^ release, and similarly did not interact with other mechanisms such as Ins(1,4,5)P_3_ and cADPR and has been studied extensively both *in vitro*^31^ and *in vivo*^42^. In EAE, **3** not only ameliorates autoimmune disease when given before the onset of disease but also after, suggesting that a compound from this class, perhaps after further optimization, may find therapeutic use in human autoimmune disease. To date, **3** as the best optimized version of **2** so far, has emerged as the most successful agent for biological applications from this work.

We used the recently reported SwissADME program^43^ to compare the drug-likeness of our three most promising compounds, **1–3**. In agreement with our initial studies, **1** was predicted to be more water soluble, but less membrane permeant compared to **2** and **3**. Unlike **1**, both **2** and **3** were predicted to cross the blood-brain barrier. All three were predicted to have good gastrointestinal absorption. Interestingly, the long alkyl chains of both **2** and **3** were highlighted as less desirable due to their increased flexibility. Predicted LogP values were 1 = −1.24, 2 = 1.52 and 3 = 1.65. The evaluation also flagged up the quaternary nitrogen, traditionally somewhat unattractive for medicinal chemistry design but, we believe at least anecdotally, to be essential to mimic the quaternary nitrogen of NAADP. Further optimization of these lead compounds may address this and future work will investigate wider SAR issues.

^1^H-NMR analysis of these pyridinium compounds showed a characteristic singlet around 5.5–5.6 ppm, representing the CH_2_ directly connected to nicotinic acid. It was also found that these pyridinum salts all contained a bromide counter ion as verified by microanalysis (Supplementary Information, Table 1). This suggested that the carboxylic acid group of nicotinic acid was protonated, rather than ionized, and that the counter ion ensures neutrality of the molecule (Fig. 8). This was investigated crystallographically for **3**.

### Crystallography

**Figure 9:**
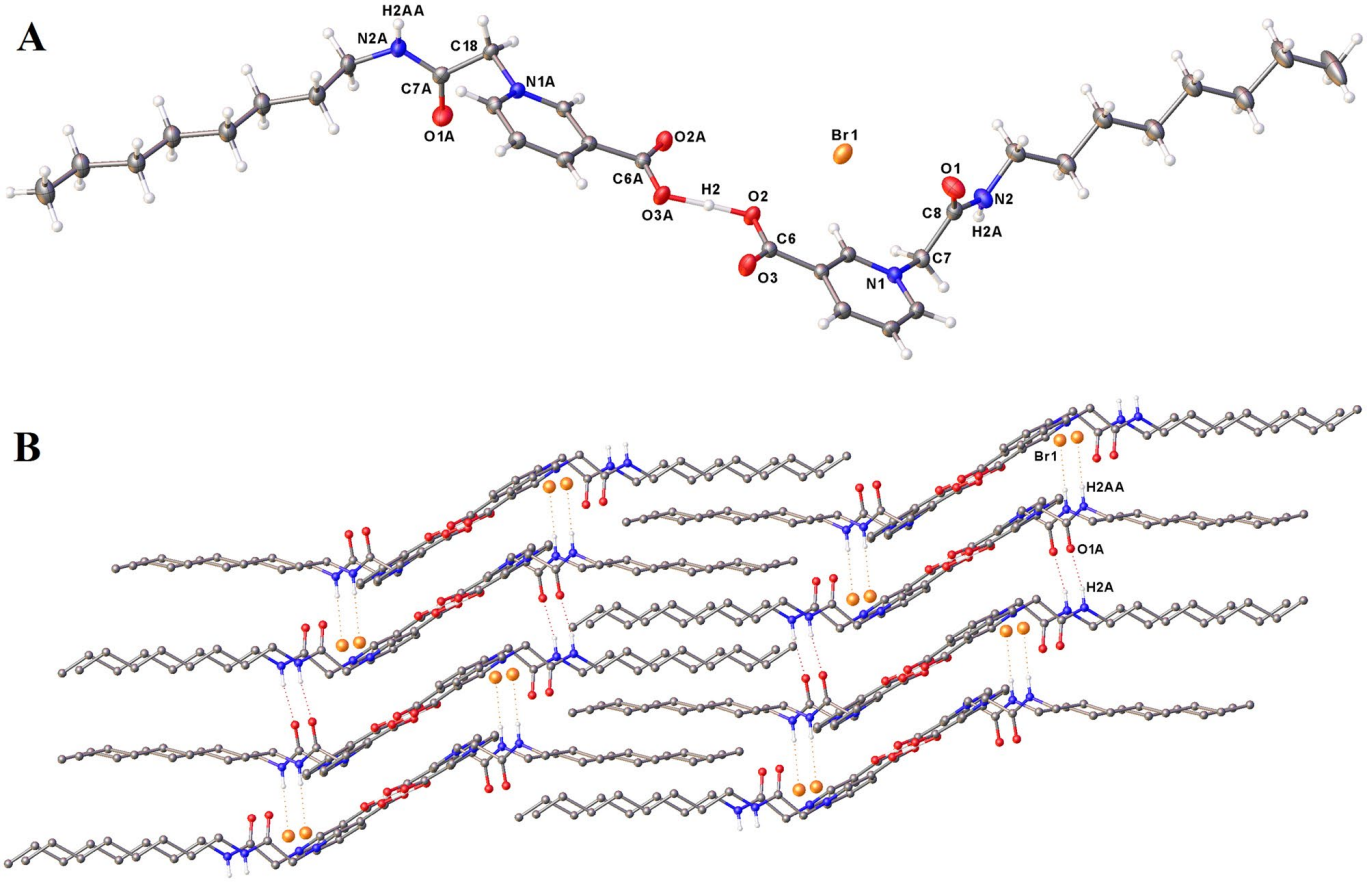
The crystal structure of **3** with ellipsoids represented at 30% probability. (**A**) The asymmetric unit; (**B**) A portion of the gross structure showing the stacking of the aromatic regions and the lipid-like alignment of the non-polar 8-carbon side chains.

X-Ray crystallography of a suitable crystal of **3** (Fig. 8) showed an asymmetric unit formed by linking O2 of the protonated species with O3 of a zwitterionic counterpart *via* hydrogen bonding (Fig. 9). Only one bromide counterion was observed for this unit, suggesting that **3** could exist as a zwitterion.

**Figure 10:**
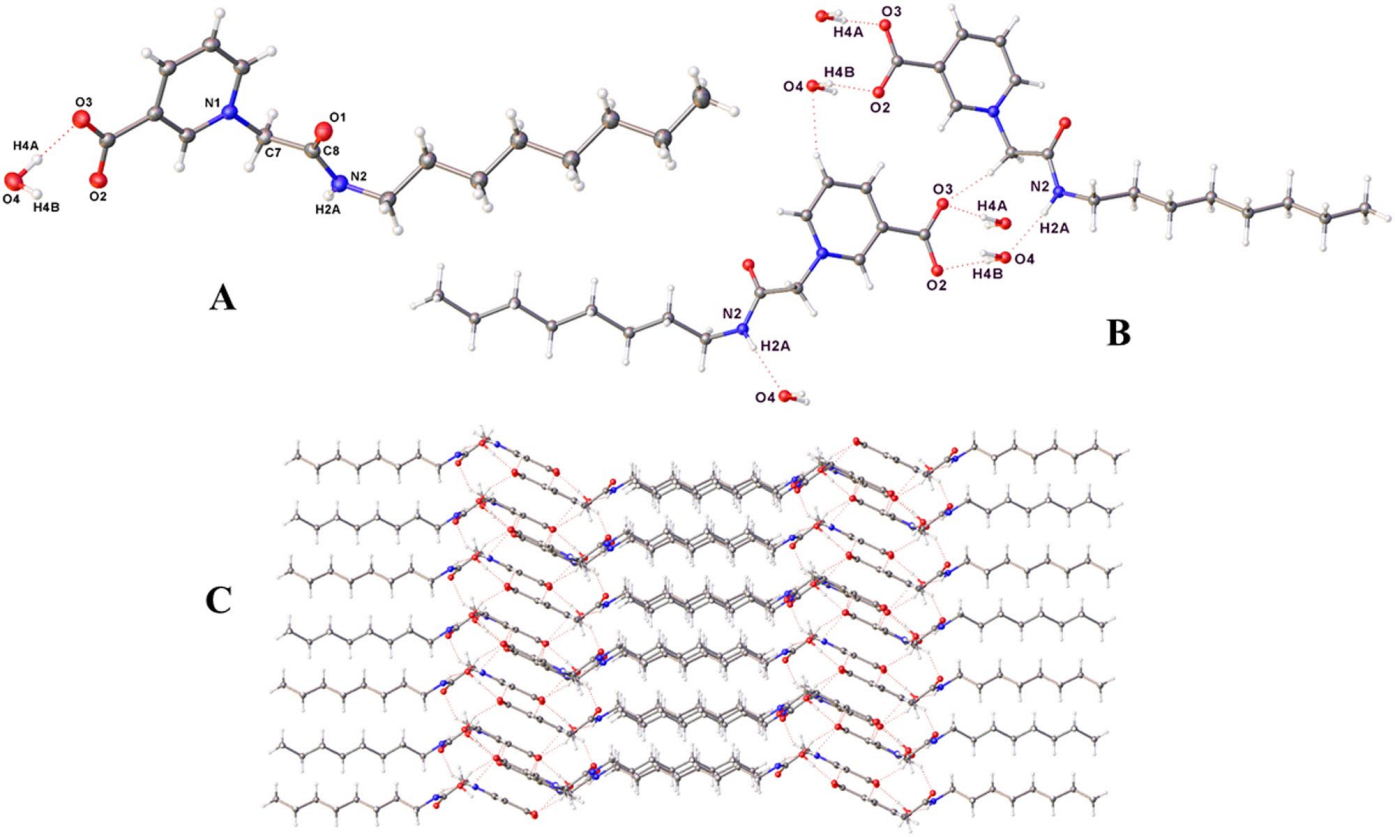
The crystal structure of **3a** with ellipsoids represented at 30% probability. (**A**) The asymmetric unit, comprising one zwitterion and one molecule of water. (**B**) Hydrogen bonding interactions. (**C**) Hydrogen-bonded sheets in the gross structure.

To study **3** in more detail, we prepared the pure zwitterionic form (**3a** (Fig. 8) by removal of the bromine counter ion. Traditional methods, including treatment with Ag(I)O to precipitate AgBr^32^, were not possible as the insolubility of the resulting zwitterion resulted in an inseparable mixture. However, treatment of **3** in methanol with DOWEX^®^ Monosphere^®^ 550 A (OH) anion exchange resin, followed by hot filtration to remove the resin afforded small white crystals of **3a**. Zwitterion **3a** is however disappointingly insoluble in H_2_O, soluble in hot MeOH and only sparingly soluble in DMSO. In contrast to **3**, that has a melting point of 195–197 °C, **3a** decomposes above 180 °C. Crystallography confirmed the integrity of the zwitterion, with the unit cell containing one molecule of the zwitterion hydrogen-bonded to one molecule of water (Fig. 10A). Hydrogen bonding between the hydrogen of the water molecule and the carboxylic acid, and the oxygen of the water and the amide N-H (Fig. 10B) generates a gross structure that is dominated by H-bonded sheets (Fig. 10C).

On balance, given the more difficult solubility and apparently better membrane permeability characteristics of **3a**, **3** seems to be the preferred compound for biological applications, with the optimal balance of solubility and membrane permeability. We have therefore not studied **3a** biologically.

The best compound in terms of potency and membrane permeability discovered in this series so far is **3**. The crystal structure study revealed that this compound presents with a bromide equivalent, crystallizes as a mixture of the zwitterionic form and free acid form that is balanced with half an equivalent of a bromide counter ion per unit cell. *In vivo* the expectation would be that it is active in the zwitterionic form, similar to that of the natural alkaloid trigonelline. **3** Has found multiple applications, being used specifically in a model of T-cell mediated autoimmunity to inhibit Ca^2+^ mobilization in intact T-cells and to attenuate downstream signalling events with relevance to autoimmune therapy;^31^ it blocked antigen-dependent increases of Ca^2+^ signals in T cells^44^ and induced a transient state of non-responsiveness in post activated effector T-cells, showing the ability to ameliorate significantly *in vivo*, both prophylactically and therapeutically, the clinical symptoms of EAE^42^. In T-cells **3** was also recently shown by intravital imaging specifically to reduce long-lasting Ca^2+^ signalling at spinal cord leptomeninges, confirming an activation checkpoint as a potential therapeutic target^45^. Finally, in a cardiovascular setting **3** blocked isoproterenol-induced diastolic Ca^2+^ transients in myocytes and almost completely prevented isoproterenol-induced cardiac arrhythmias *in vivo*, indicating a pivotal role for NAADP/Ca^2+^ signalling in excitation-contraction coupling and uncovering a new target for antiarrhythmic therapy^46^.

## Conclusion

In summary, we present for the first time a synthetic route to stable, membrane-permeant NAADP fragment analogues, the best of which are effective inhibitors of NAADP-induced Ca^2+^ release in T-lymphocytes. This also provides the wider synthetic context in which to view compound **3** BZ194, data for which have already been published^31^. These can be used as molecular tools to investigate and modulate NAADP/Ca^2+^ signalling. Lead compound **2** showed positive and specific effects on Ca^2+^ signalling *in vitro* and *in vivo* in an EAE model of multiple sclerosis; it showed beneficial effects towards autoimmune disease, suggesting that further modification of such compounds may lead to potential therapeutic agents. Another compound from this series **3** has also been investigated *in vitro* and *in vivo*, demonstrating the NAADP/Ca^2+^ signalling pathway as a potential novel target in autoimmunity^31,42,44,45^ and for ventricular cardiomyocyte arrhythmias^46^. Thus, suitable inhibitors of NAADP/Ca^2+^ signalling might potentially be further developed for clinical use. The fact that these active compounds were developed through evaluation of only a relatively small library of compounds indicate that more optimized lead compounds, perhaps with better potency and more fine-tuned physicochemical properties, can likely be discovered in future work with implications for therapeutic intervention.

## Experimental

### General

NAADP was supplied by Sigma. cADPR and Ins(1,4,5)P_3_ were obtained from Biolog (Bremen, Germany). All Lewis and DA rats were bred in the animal facility of the Max Planck Institute of Neurobiology (Martinsried, Germany) and all experiments were conducted according to the guidelines of The Committee on Animals of the Max Planck Institute of Neurobiology and were approved by the Regierung von Oberbayern. All reagents and solvents were of commercial quality and were used directly unless otherwise described. ^1^H NMR and ^13^C NMR were collected in *d*_6_-DMSO or D_2_O, either on a JEOL Delta at 270 MHz (^1^H) and 68 MHz (^13^C) or on a Varian Mercury-vx machine at 400 MHz (^1^H) and 100 MHz (^13^C). Abbreviations for splitting patterns are described below: s (singlet); d (doublet); t (triplet) and m (multiplet) etc. Low resolution FAB mass spectra were recorded on a Micromass Autospec instrument on samples in a *m*-nitrobenzyl alcohol matrix at the Mass Spectrometry Centre, University of Bath. Accurate mass spectra were recorded either by the Mass Spectrometry Centre at the University of Bath or by the ZX 90Q Mass Spectrometry Service, Department of Pharmacy and Pharmacology, Bath. HPLC analysis was carried out on a Waters 2695 Alliance module equipped with a Photodiode array detector and a XTerra MSC_18_ 3.5 μm (4.6 × 150 mm) column. All samples were eluted with a gradient of MeCN against H_2_O (component as specified) at 1 mL/min and monitored at 254 nm.

### Microinjection into Jurkat T cells

Microinjections were carried out as described. Briefly, an Eppendorf system was used (transjector type 5246, micromanipulator type 5171, Eppendorf-Netheler-Hinz, Hamburg, Germany) with Femtotips II as pipettes. NAADP was diluted to its final concentration in intracellular buffer (20 mM HEPES, 110 mM KCl, 2 mM MgCl_2_, 5 mM KH_2_PO_4_, 10 mM NaCl, pH 7.2) and filtered (0.2 μm) before use. Injections were made using the semi-automatic mode of the system with the following instrumental settings: injection pressure 60 hPa, compensatory pressure 30 hPa, injection time 0.3–0.5 s and velocity of the pipette 700 μm/s. Under such conditions the injection volume was 1–1.5% of the cell volume

### Ca^2+^ measurements in intact T cell suspensions

Intact Jurkat T-lymphocytes were loaded with fura2/AM. Ratiometric determination of [Ca^2+^]_i_ was carried out in cell suspension in a Hitachi F2000 fluorimeter at room temperature at excitation wavelengths of 340 and 380 nm (alternating) and an emission wavelength of 495 nm. Each experiment was calibrated by addition of Triton X100 (10% v/v final concentration) to obtain the maximal ratio and subsequent addition of 4 mM EGTA/40 mM Tris-base to obtain the minimal ratio.

### *In vitro* proliferation assay (rat encephalitogenic T cells)

Encephalitogenic T cells specific for the myelin protein myelin basic protein were established as described previously. The cells were retrovirally engineered to express green fluorescent protein (T_MBP-GFP_ cells)^40^. Resting T_MBP-GFP_ cells were plated in 96- wells (5 × 10^4^ /well) and stimulated by the addition of thymocytes (1.5 × 10^6^ / well) as antigen-presenting cells and MBP (5 µg/mL) as specific antigen, or ConA (2 µg/mL). After 2 days [^3^H]-thymidine (*Amersham Biosciences*) was added to the activated cells (final concentration 4 µCi/ml); after 16 h of incubation [^3^H]-thymidine incorporation was measured (Matrix 9600- Direct Beta Counter *Packad*) as previously reported^31^.

### *In vitro* toxicity assay (rat encephalitogenic T cells)

Resting T_MBP-GFP_ cells were plated in 96- wells (5 × 10^4^ /well). Substances to test were added at different concentration to the cells and incubated for 1 h. Cells stimulated by the addition of thymocytes (1.5 × 10^6^ / well) and MBP. Absolute numbers of T_MBP-GFP_ cells were determined by quantitative cytofluorometrical analysis after 24 and 48 hours, relative to known amounts of added PE-fluorescent beads, prior to appropriate morphological gating for live/dead cells (FACS-Calibur *BektonDickinson*)^31^.

### EAE induction and *in vivo* toxicity assay (Lewis rat)

Nicotinic acid or **2** was injected i.p. to recipient healthy Lewis rats and to EAE-induced rats twice a day for 6 days at the following concentrations: 100 µM (15 µmol substance /150 g body weight) and 500 µM (75 µmol substance /150 g body weight). To induce EAE, transfer of encephalitogenic T cells were performed as follows: 5 × 10^4^ activated T_MBP-GFP_ cells were transferred i.v. into recipient Lewis rats on day 0. Clinical EAE was graded in five scores: 0.5, loss of tail tonus; 1, tail paralysis; 2, gait disturbance; 3, hind limb paralysis; 4, tetraparesis; 5, death^31^. The animals’ weight and clinical scores were measured twice a day. Rats were sacrificed after 7 days and spleen, spinal cord and parathymic lymph nodes were extracted, for analysis of organ derived cell suspensions. Absolute numbers of T_MBP-GFP_ cells in the organs were determined by quantitative cytofluorometrical analysis (FACS-Calibur *BektonDickinson*)^31^.

### X-Ray crystallography

Data for **3** were obtained at 150 K using a Nonius KappaCCD diffractometer and Mo-Kα radiation. Solution and refinement of the model were effected using the SHELX^47,48^ suite of programs *via* Olex-2^49^. Crystal Data for C_16_H_24.5_N_2_O_3_Br_0.5_ (3): *M* = 332.83 g mol^−1^, triclinic, space group *P*-1 (no. 2), *a* = 8.7780(1), *b* = 9.2460(1), *c* = 21.3480(4) Å, *α* = 91.924(1), *β* = 99.210(1), *γ* = 102.398(1)°, *U* = 1666.26(4) Å^3^, *Z* = 4, *T* = 150 K, *μ*(Mo-Kα) = 1.281 mm^−1^, *D*_calc_ = 1.327 g cm^−3^, 25522 reflections measured (7.616° ≤ 2θ ≤ 54.964°), 7559 unique (*R*_int_ = 0.0404) which were used in all calculations. The final *R*_1_ was 0.0321 (*I* > 2σ(*I*)) and *wR*_2_ was 0.0820 (all data). The asymmetric unit in the structure was seen to comprise one cation, and one bromide anion (Fig. 9A). The cation presented as a dimer of two carboxylates, linked by a single proton (H2), located between O2 and O3A, which was refined without restraints [O2-H2 1.18(3), O3A-H2 1.27(3), O2…O3A 2.457(2)Å; O2-H2-O3A 178(3)°]. The nitrogen bound hydrogens were also located and, in both cases, refined at a distance of 0.89 Å from the relevant parent atom. H2A (attached to N2), is involved in hydrogen-bonding to O1A of a lattice neighbour, while H2AA (attached to N2A) similarly interacts with a bromide anion. [N2-H2A 0.886(5), H2A…O1A 1.96(1), N2…O1A 2.834(2)Å; N2-H2A-O1A 168(2)° N2A-H2AA 0.887(5), H2AA…Br1 2.62(1), N2A…Br1 3.436(1)Å; N2A-H2AA-Br1 153(2)°]. A portion of the gross structure is illustrated in Fig. 9B which shows the hydrogen-bonding interactions and the alignment of the interdigitated non-polar 8-carbon side chains.

A suitable crystal of **3a** was selected and mounted on an Agilent SuperNova, Dual, Cu at zero, EosS2 diffractometer. The crystal was kept at 150.00(10) K during data collection. Using Olex2^49^, the structure was solved with the ShelXS^48^ structure solution program using Direct Methods and refined with the ShelXL^47^ refinement package using Least Squares minimization. Crystal Data for C_16_H_26_N_2_O_4_ (3a): (*M* = 310.39 g/mol): monoclinic, space group *P*2_1_/c (no. 14), *a* = 21.6725(5) Å, *b* = 10.3908(2) Å, *c* = 7.22786(17) Å, *β* = 98.845(2)°, *V* = 1608.32(7) Å^3^, *Z* = 4, *μ*(CuKα) = 0.750 mm^−1^, *D*_calc_ = 1.282 g cm^−3^, 12941 reflections measured (8.258° ≤ 2Θ ≤ 146.2°), 3201 unique (*R*_int_ = 0.0352, *R*_sigma_ = 0.0277) which were used in all calculations. The final *R*_1_ was 0.0413 (*I* > 2σ(*I*)) and *wR*_2_ was 0.1148 (all data). The asymmetric unit comprises one zwitterion and a molecule of water (Fig. 10A). Fig. 10B shows the hydrogen-bonded interactions and Fig. 10C a portion of the gross structure.

### Synthesis of bromoacetylamides (protocol A)

To a stirred solution of 2-bromoacetyl bromide (12 mmol) in ether (10 mL) in an ice-salt bath was added dropwise a solution of amines (24 mmol) in ether (10 mL) over 30 mins. The resulting clear solution was stirred in ice-salt bath for 15–30 mins and then quenched by addition of cold water (20 mL). The organic layer was separated and washed successively with hydrochloride solution (1 M, 2 × 20 mL), NaOH solution (1 M, 2 × 20 mL) and brine (2 × 20 mL). The solvent was removed and the residue was dried *in vacuo* and used in next step without further purification or purified by flash column chromatography as specified.

### Procedure for alkylation reaction (protocol B)

Nicotinic acid (1.62 mmol) and 2-bromoacetylamides (1.62 mmol) were dissolved in dry DMF (4 mL) and the reaction solution was heated at 60–70 °C for 16 h. DMF was evaporated *in vacuo* and the resulting residue was dissolved in small amount of MeOH. The desired compounds were precipitated by dropwise addition of ether.

### 3-Carboxy-1-diethylcarbamoylmethyl-pyridinium bromide (18)

2-Bromoacetyl bromide and diethylamine were reacted under general protocol A to afford **4** as a colourless oil (1.39 g, 60%). ^1^H NMR (CDCl_3_, 270 MHz) δ 3.73 (s, 2H, CH_2_CO) 3.25 (q, *J* = 7.5 Hz, 4H, CH_2_ CH_3_), 1.24 (t, *J* = 7.5 Hz, 3H, CH_3_) and 1.03 (t, *J* = 7.5 Hz, 3H, CH_3_) ppm. Nicotinic acid (1.62 mmol) and 2-bromo-*N*, *N*-diethylacetamide 4 (1.62 mmol) were reacted under general protocol B to afford **18** as a yellow oil (0.34 mmol, 21%); ^1^H NMR (D_2_O, 270 MHz) δ 9.06 (m, 1 H, H_N_-2), 8.87 (m, 1 H, H_N_-6), 8.74 (m, 1 H, H_N_-4), 8.03 (m, 1 H, H_N_-5), 5.68 (s, 2H, CH_2_CO), 3.33 (q, 4H, *J* = 7.5 Hz, 2 × CH_2_), 1.19 (t, 3H, *J* = 7.5 Hz, CH_3_) and 1.00 (t, 3H, *J* = 7.5 Hz, CH_3_) ppm; *m/z* (FAB^+^) 237.0 [(M)^+^, 100%]; HRMS (FAB^+^) calcd. for C_12_H_17_N_2_O_3_^+^ 237.1239 [M]^+^, found 237.1238.

### 1-butylcarbamoylmethyl-pyridinium bromide (19)

2-Bromoacetyl bromide and *n*-butylamine were reacted under general protocol A to afford **5** as a low melting point solid (1.77 g, 76%). mp: <50 °C; ^1^H NMR (CDCl_3_, 270 MHz) δ 6.65 (brs, 1H, NH), 3.81 (s, 2H, CH_2_CO), 3.19 (m, 2H, CH_2_), 1.41 (m, 2H, CH_2_), 1.25 (m, 2H, CH_2_) and 0.78 (m, 3H, CH_3_). Nicotinic acid (1.62 mmol) and 2-bromo-*N*-butylacetamide 5 (1.62 mmol) were reacted under general protocol B to afford **19** as a white solid (447 mg, 87%); mp: 190–194 °C; ^1^H NMR (270 MHz, D_2_O) δ 9.25 (s, 1H, H_N_-2), 8.96 (d, *J*_6,5_ = 5.3 Hz, 1H, H_N_-6), 8.85 (d, *J*_4,5_ = 5.9 Hz, 1H, H_N_-4), 8.13 (dd, *J*_5,4_ = 5.9 and *J*_5,6_ = 5.3 Hz, 1H, H_N_-5), 5.43 (s, 2H, CH_2_CO), 3.16 (m, 2H, CH_2_), 1.40 (m, 2H, CH_2_), 1.20 (m, 2H, CH_2_) and 0.77 (m, 3H, CH_3_) ppm; *m/z* (FAB^+^) 237.0 [(M)^+^, 100%]; HRMS (FAB^+^) calcd. for C_12_H_17_N_2_O_3_^+^ 237.1239 [M]^+^, found 237.1240; *t*_*R*_ = 4.6 mins (solvent: 5–50% MeCN against H_2_O over 30 mins). Anal. Calcd for C_12_H_17_N_2_O_3_Br: C, 45.44; H, 5.40; N, 8.83. Found: C, 44.60; H, 5.26; N, 8.56.

### Carboxyl-1-phenylcarbamoylmethyl-pyridinium bromide (20)

2-Bromoacetylamine and aniline were reacted under general protocol A to afford **6** as a yellow solid (1.82 g, 71%); mp: 128–131 °C; ^1^H NMR (CDCl_3_, 270 MHz) δ 8.13 (brs, 1H, NH) 7.52 (d, *J* 2.7 Hz, 2H, ArH-2 and ArH-6), 7.34 (m, 2H, ArH-3 and ArH-5), 7.17 (m, 1H, ArH-4) and 4.00 (s, 2H, CH_2_CO) ppm; ^13^C NMR (CDCl_3_, 100.5 MHz) δ 163.3, 137.1 (both C), 129.3, 125.4, 120.3 (all CH), 29.9 (CH_2_) ppm; *m/z* (FAB^+^) 213.9 [(M + H)^+^,100%]; HRMS (FAB^+^) calcd. for C_8_H_9_^79^BrNO^+^ 213.9868 [M + H]^+^, found 213.9858; calcd for C_8_H_9_^81^BrNO^+^ 215.9847 [M + H]^+^, found 215.9839. Nicotinic acid (1.62 mmol) and 2-bromo-*N*-phenylacetamide **6** (1.62 mmol) were reacted under general protocol B to afford** 20** as a white solid (454 mg, 83%); mp: ^1^H NMR (DMSO, 270 MHz) δ 10.85 (brs, 1H, NH), 9.52 (s, 1H, H_N_-2), 9.14 (m, 1H, H_N_-6), 9.09 (m, 1H, H_N_-4), 9.00 (m, 1H, H_N_-5), 7.62–7.08 (m, 5 H, 5 × ArH) and 5.76 (s, 2H, CH_2_CO) ppm; ^13^C NMR (DMSO, 100 MHz) δ 163.8, 163.5, 146.2, 138.9 (all C), 148.2, 147.9, 129.6, 127.9, 124.6, 119.8 (all CH) and 62.9 (CH_2_) ppm; *m/z* (FAB^+^) 257.1 [(M]^+^, 100%]; HRMS (FAB^+^) calcd. for C_14_H_13_N_2_O_3_^+^ 257. 0926 [M]^+^, found 257.0933; *t*_*R*_ = 6.6 mins (solvent: 5–50% MeCN against H_2_O over 30 mins).

### 3-Carboxy-1-[(4-ethyl-phenylcarbamoyl)-methyl]-pyridinium bromide (21)

2-Bromoacetyl bromide and 4-ethyl-aniline were reacted under general protocol A to afford **7** as a yellow solid (2.12 g, 73%); ^1^H NMR (CDCl_3_, 270 MHz) δ 8.07 (brs, 1H, NH), 7.40 (d, *J* = 8.4 Hz, 2H, 2 × ArH), 7.15 (d, *J* = 8.4 Hz, 2H, 2 × ArH), 4.00 (s, 2H, CH_2_CO), 2.59 (q, *J* = 7.7 Hz, 2H, CH_2_) and 1.18 (t, *J* = 7.7 Hz, 3H, CH_3_) ppm; *m/z* (FAB^+^) 242.0 [(M + H)^+^, 100%], 244.0 [(M + H)^+^, 93%]; HRMS (FAB^+^) calcd. for C_10_H_13_^79^BrNO^+^ 242.0181 [M + H]^+^, found 242.0182; calcd for C_10_H_13_^81^BrNO^+^ 244.0160 [M + H]^+^, found 244.0174. Nicotinic acid (1.62 mmol) and 7 (1.62 mmol) were reacted under general protocol B to afford **21** as a white solid (461 mg, 78%); mp: 210–214 °C; ^1^H NMR (D_2_O, 270 MHz) δ 9.29 (s, 1H, H_N_-2), 8.98 (d, *J*_6,5_ = 8.7 Hz,1H, H_N_-6), 8.91 (d, *J*_4,5_ = 6.5 Hz, 1H, H_N_-4), 8.14 (dd, *J*_5,6_ = 8.7 Hz and *J*_5,4_ = 6.5 Hz, 1H, H_N_-5), 7.28 (d, *J* = 8.1 Hz, 2H, ArH-2 and ArH-6), 7.18 (d, *J* = 8.1 Hz, 2H, ArH-3 and ArH-5), 5.60 (s, 2H, CH_2_CO), 2.49 (q, *J* = 7.7 Hz, 2H, CH_2_) and 1.06 (t, *J* = 7.7 Hz, 3H, CH_3_) ppm; ^13^C NMR (DMSO, 100 MHz) δ 163.6, 163.4, 140.0, 136.5, 131.1 (all C); 149.9, 148.3, 146.7, 128.8, 128.4, 119.9 (all CH); 63.1, 28.5 (both CH_2_) and 16.5 (CH_3_) ppm; *m/z* (FAB^+^) 285.1 [(M)^+^, 100%]; HRMS (FAB^+^) calcd. for C_16_H_17_N_2_O_3_^+^ 285.1239 [M]^+^, found 285.1239. Anal. Calcd for C_16_H_17_N_2_O_3_Br: C, 52.62; H, 4.69; N, 7.67. Found: C, 52.40; H, 4.63; N, 7.38.

### 3-Carboxy-1-cyclohexylcarbamoylmethyl-pyridinium bromide (22)

2-Bromoacetyl bromide and cyclohexylamine were reacted under general protocol A to afford **8** as a white fine powder (2.38 g, 90%); mp: 109–111 °C (recrystallized from hexane); ^1^H NMR (CDCl_3_, 270 MHz) δ 6.33 (brs, 1H, NH), 3.84 (s, 2H, CH_2_CO), 3.74 (m, 1H, CHN) and 1.10–1.92 (m, 10 H, ring-H) ppm; ^13^C NMR (CDCl_3_, 100 MHz) δ 164.7 (C), 49.6 (CH), 33.4, 30.2, 26.1 and 25.3 (all CH_2_) ppm; m/z (FAB^+^) 220.0 [(M + H)^+^, 70%]; 222.0 [(M + H)^+^, 70%]; HRMS (FAB^+^) calcd. for C_8_H_15_^79^BrNO 220.0337 [M + H]^+^, found 220.0333; calcd C_8_H_15_^81^BrNO^+^ 222.0317[M + H]^+^, found 222.0317. Nicotinic acid (1.62 mmol) and 2-bromo-*N*-cyclohexylacetamide 8 (1.62 mmol) were reacted under general protocol B to afford **22** as a white solid (521 mg, 94%); mp: 188–192 °C; ^1^H NMR (DMSO, 270 MHz) δ 9.52 (s, 1H, H_N_-2), 9.17 (d, 1H1H, *J*_6,5_ = 6.4 Hz, H_N_-6), 9.03 (d, 1H, *J*_4,5_ = 8.2 Hz, H_N_-4), 8.55 (d, *J* = 7.7 Hz, NH) 8.30 (dd, *J*_5,6_ = 8.2 and *J*_5,4_ = 6.4 Hz, 1H, H_N_-5), 5.52 (s, 2H, CH_2_CO), 3.57 (m, 1H, CH) and 1.80–1.24 (m, 10 H, ring-H) ppm; ^13^C NMR (DMSO, 100 MHz) δ 163.6, 163.5, 131.0 (all C), 149.7, 148.0, 146.4, 128.3, 39.7 (all CH), 62.5, 33.1, 26.0 and 25.2 (all CH_2_) ppm; *m/z* (FAB^+^) 263.3 [(M)^+^, 100%]; *t*_*R*_ = 2.9 mins (solvent: a gradient of 20–95% MeCN against H_2_O over 25 mins). Anal. Calcd for C_14_H_17_N_2_O_3_Br: C, 48.99; H, 5.58; N, 8.16. Found: C, 48.90; H, 5.46; N, 8.04.

### 3-Carboxy-1-[(2-bromo-phenylcarbamoyl)-methyl]-pyridinium bromide (23)

2-Bromoacetyl bromide and 2-bromo aniline were reacted under general protocol A. The crude product was purified by flash column chromatography, eluted with DCM-Hexane 10:1 v/v to afford **9** as a yellow solid (1.83 g, 52%); mp: 96–98 °C; ^1^H NMR (CDCl_3_, 270 MHz) δ 8.15 (brs, 1H, NH), 7.77 (m, 1H, ArH-3), 7.43 (d, *J* = 7.8 Hz, 1H, ArH-6), 7.23 (m, 2H, ArH-4 and ArH-5) and 4.00 (s, 2H, CH_2_CO) ppm; ^13^C NMR (CDCl_3,_ 100 MHz) δ 164.3, 138.1, 122.6 (all C), 130.3, 128.2, 123.0, 118.5 (all CH) and 29.3 (CH_2_) ppm; *m/z* (FAB^+^) 293.9 [(M + H)^+^, 100%]. Nicotinic acid (1.62 mmol) and 2-bromo-*N*-(2-bromo-phenyl)-acetamide **9** (1.62 mmol) were reacted under general protocol B to afford **23** as a white solid (544 mg, 81%); mp: 216–218 °C; ^1^H NMR (D_2_O, 270 MHz) δ 9.36 (s, 1H, H_N_-2), 9.04 (d, *J*_6,5_ = 8.1 Hz, 1H, H_N_-6), 8.99 (d, *J*_4,5_ = 5.6 Hz, 1H, H_N_-4), 8.23 (dd, *J*_5,6_ = 8.1 and *J*_5,4_ = 5.6 Hz, 1H, H_N_-5), 7.68 (s, 1H, ArH-3), 7.31 (m, 3H, ArH-4, ArH-5 and ArH-6) and 5.70 (s, 2H, CH_2_CO) ppm, ^13^C NMR (DMSO_6,_ 100 MHz) δ 164.2, 163.5, 139.9, 131.1, 121.6 (all C), 149.9, 148.3, 146.1, 131.5, 127.8, 126.7, 121.1, 116.0 (all CH) and 62.5 (CH_2_) ppm; *m/z* (FAB^+^) 335.1 [(M + H)^+^, 100%]. *t*_*R*_ = 4.9 mins (solvent: a gradient of 20–95% MeCN against H_2_O over 25 mins).

### 3-Carboxy-1-dibutylcarbamoylmethyl-pyridinium bromide (2)

2-Bromoacetyl bromide and dibutylamine were reacted under general protocol A to afford **10** as a colourless oil (2.30 g, 76%); ^1^H NMR (CDCl_3_, 270 MHz) δ 3.77 (m, 2H, CH_2_CO), 3.23 (m, 4H, 2 × CH_2_N), 1.48 (m, 4H, 2 × CH_2_), 1.25 (m, 4H, 2 × CH_2_) and 0.86 (m, 6 H, 2 × CH_3_) ppm; ^13^C NMR (CDCl_3_, 100.5 MHz) δ 166.3 (C), 49.0, 46.2, 31.6, 29.7, 27.0, 20.5 (all CH_2_), 14.3 and 14.2 (both CH_3_) ppm; *m/z* (FAB^+^) 252.0 [(M + H)^+^, 95%], 250.0 [(M + H)^+^, 100%], 170 [(M-Br)^+^, 12%]; HRMS (FAB^+^) calcd. for C_10_H_21_^79^BrNO^+^ 250.0807 (M + H)^+^, found 250.0798; calcd for C_10_H_21_^81^BrNO^+^ 252.0786 [M + H]^+^, found 252.0783. Nicotinic acid (1.62 mmol) and 2-bromo-*N*, *N*-dibutylacetamide **10** (1.62 mmol) were reacted under general protocol B. The crude product was purified by flash chromatography, eluted with a gradient of 0–5% methanol in DCM to afford **2** as a yellow oil (483 mg, 80%). ^1^H NMR (D_2_O, 270 MHz) δ 9.26 (s, 1H, H_N_-2), 9.10 (m, 1H, H_N_-6), 8.90 (m, 1H, H_N_-4), 8.24 (m, 1H, H_N_-5), 5.82 (s, 2H, COCH_2_), 3.44 (m, 4H, 2 × CH_2_), 1.72 (m, 2H, CH_2_), 1.54 (m, 2H, CH_2_), 1.40 (m, 2H, CH_2_), 1.30 (m, 2H, CH_2_) and 0.92 (m, 6 H, 2 × CH_3_) ppm; ^13^C NMR (D_2_O, 100 MHz) δ 164.3, 163.6, 131.0 (all C), 149.9, 148.20, 146.5, 128.3 (all CH), 62.0, 47.3, 46.6, 30.9, 30.1, 20.5, 20.4 (all CH_2_), 14.7 and 14.6 (both CH_3_) ppm; *m/z* (FAB^+^) 293.0 [(M)^+^, 100%]; HRMS (FAB^+^) calcd. for C_16_H_25_N_2_O_3_^+^ 293.1865 [M]^+^, found 293.1876. *t*_*R*_ = 5.3 mins (solvent: a gradient of 20–95% MeCN against H_2_O over 25 mins).

### 3-Carboxy-1-dipropylcarbamoylmethyl-pyridinium bromide (24)

2-Bromoacetyl bromide and dipropylamine were reacted under general protocol A to afford **11** as a yellow oil (1.72 g, 65%); ^1^H NMR (CDCl_3_, 270 MHz) δ 3.81 (s, 2H, CH_2_CO), 3.24 (m, 4H, 2 × CH_2_), 1.55 (m, 4H, 2 × CH_2_) and 0.86 (m, 6 H, 2 × CH_3_) ppm; ^13^C NMR (CDCl_3_, 100 MHz) δ 166.5 (C), 50.4, 47.7, 26.2, 22.2, 20.4 (all CH_2_) and 11.2 (CH_3_) ppm; *m/z* (FAB^+^) 222.0 [(M + H)^+^, 100%], 224.0 [(M + H)^+^, 90%]. Nicotinic acid (1.62 mmol) and 2-bromo-*N*,*N*-dipropylacetamide **11** (1.62 mmol) were reacted under general protocol B to afford **24** as a yellow oil (252 mg, 45%); ^1^H NMR (D_2_O, 270 MHz) δ 9.24 (s, 1H, H_N_-2), 9.04 (d, *J*_6,5_ = 8.1 Hz, 1H, H_N_-6), 8.87 (d, *J*_4,5_ = 5.1 Hz, 1H, H_N_-4), 8.20 (dd, *J*_5,6_ = 8.1 and *J*_5,4_ = 5.1 Hz, 1H, H_N_-5), 5.80 (s, 2H, CH_2_CO), 3.34 (m, 4H, 2 × CH_2_), 1.71 (m, 2H, CH_2_), 1.54 (m, 2H, CH_2_), 0.92 (t, *J* = 7.4 Hz, CH_3_) and 0.82 (t, *J* = 7.4 Hz, CH_3_) ppm; ^13^C NMR (D_2_O, 100 MHz) δ 164.7, 164.6, 132.1 (all C), 148.6, 147.4, 146.8, 128.1 (all CH), 62.1, 49.4, 48.9, 21.41, 20.38 (all CH_2_), 10.9 and 10.8 (both CH_3_) ppm; *m/z* (FAB^+^) 266.3 [(M + H)^+^, 100%]; HRMS (FAB^+^) calcd. for C_14_H_21_N_2_O_3_^+^ 265.1552 [M]^+^, found 265.1556. *t*_*R*_ = 3.0 mins (solvent: a gradient of 20–95% MeCN against H_2_O over 25 mins).

### 3-Carboxy-1-decylcarbamoylmethyl-pyridinium bromide (25)

2-Bromoacetyl bromide and *n*-decylamine were reacted under general protocol A. The crude product was purified by flash column chromatography, eluted with DCM-hexane 10:1 v/v to afford **12** as a colourless oil (2.72 g, 81%); ^1^H NMR (CDCl_3_, 270 MHz) δ 6.49 (s, brs, 1H, NH), 3.86 (s, 2H, CH_2_CO), 3.25 (m, 2H, CH_2_), 1.52 (m, 2H, CH_2_), 1.24 (m, 14H, 7 × CH_2_) and 0.86 (m, 3H, CH_3_) ppm; ^13^C NMR (CDCl_3_, 67.5 MHz) δ 165.4 (C), 40.7, 32.3, 29.9, 29.8, 29.7, 29.67, 29.65, 27.2, 23.1 (all CH_2_) and 14.6 (CH_3_) ppm; *m/z* (FAB^+^) 278.1 [(M + H)^+^, 98%], 279.1 [(M + H)^+^, 91%]; HRMS (FAB^+^) calcd. for C_12_H_25_^79^BrNO 278.1120 [M + H]^+^, found 278.1120; calcd. for C_12_H_25_^81^BrNO 280.1099 [M + H]^+^, found 280.1103. Nicotinic acid (1.62 mmol) and 2-bromo-*N*-decylacetamide **12** (1.62 mmol) were reacted under general protocol B to afford **25** as a white solid (625 mg, 96%); mp: 185–186 °C; ^1^H NMR (DMSO, 270 MHz) δ 9.53 (s, 1H, H_N_-2), 9.19 (d, *J*_6,5_ = 8.1 Hz, 1H, H_N_-6), 9.03 (d, *J*_4,5_ = 4.6 Hz, 1H, H_N_-4), 8.70 (brs, 1H, NH), 8.31 (m, 1H, H_N_-5), 5.57 (s, 2H, CH_2_CO), 3.13 (m, 2H, CH_2_), 1.24–1.61 (m, 16 H, 8 × CH_2_) and 0.83 (m, 3H, CH_3_) ppm; ^13^C NMR (DMSO, 100 MHz) δ 164.5, 163.6, 131.1 (all C), 149.7, 148.0, 146.4, 128.3 (all CH), 62.4, 32.2, 29.9, 29.7, 29.7, 29.6, 27.3, 23.0 (all CH_2_) and 14.9 (CH_3_) ppm; HRMS (FAB^+^) calcd. for C_18_H_29_N_2_O_3_^+^ 321.2178 [M]^+^, found 321.2166; *t*_*R*_ = 4.2 mins (solvent: a gradient of 35–95% MeCN against H_2_O over 25 mins). Anal. Calcd for C_18_H_29_N_2_O_3_Br: C, 53.87; H, 7.28; N, 6.98. Found: C, 54.00; H, 7.30; N, 6.99.

### 3-Carboxy-1-octylcarbamoylmethyl-pyridinium bromide (3)

The title compound was synthesized and characterized as described fully elsewhere^31^ and was crystallized from MeOH/acetone giving **3** as needles; mp: 195–197 °C; ^1^H NMR (DMSO, 270 MHz) δ 9.45 (s, 1H, H_N_-2), 9.07 (s, *J*_6,5_ = 5.9 Hz, 1H, H_N_-6), 8.94 (s, *J*_4,5_ = 8.2 Hz, 1H, H_N_-4), 8.51 (m, 1H, NH), 8.22 (dd, *J*_5,4_ = 8.2 and *J*_5,6_ = 5.9 Hz, 1H, H-5), 5.45 (s, 2H, CH_2_CO), 3.03 (m, 2H, CH_2_), 1.39–1.18 (m, 12H, 6 × CH_2_) and 0.78 (m, 3H, CH_3_) ppm; ^13^C NMR (DMSO, 100 MHz) δ 164.4, 163.5, 130.9 (all C), 149.7, 147.9, 146.4, 128.1 (all CH), 62.1, 31.7, 29.3, 29.2, 29.1, 26.8, 22.6 (all CH_2_) and 14.5 (CH_3_) ppm; HRMS (FAB^+^) calcd. for C_16_H_25_N_2_O_3_^+^ 293.1865 [M]^+^, found 293.1870. Anal. Calcd for C_16_H_25_N_2_O_3_Br: C, 51.48; H, 6.75; N, 7.50. Found: C, 50.60; H, 6.76; N, 7.36.

### 3-Carboxy-1-octylcarbamoylmethyl-pyridinium (3a)

To a solution of** 3** (100 mg) in MeOH (1 mL) was added DOWEX^®^ Monosphere^®^ 550 A (OH) anion exchange resin. After 30 min, a white precipitate had formed, MeOH (1 mL) was added and the suspension heated to 50 °C in a water bath to dissolve the precipitate. The resin was removed from the methanolic solution by hot filtration and the filtrate allowed to cool. Small white crystals of **3a** formed and were used for crystallography; mp: decomp. ≥180 °C; ^1^H NMR (DMSO, 270 MHz) δ 9.22 (s, 1H, H_N_-2), 8.87 (d, *J*_6,5_ = 6.1 Hz, 1H, H_N_-6), 8.84 (d, *J*_4,5_ = 7.9 Hz, 1H, H_N_-4), 8.72–8.70 (m, 1H, NH), 8.07 (dd, *J*_5,4_ = 7.9 and *J*_5,6_ = 6.1 Hz, 1H, H-5), 5.47 (s, 2H, CH_2_CO), 3.13 (dt, *J* = 6.8, 6.9 Hz, 2H, CH_2_), 1.47–1.41 (m, 2H, CH_2_) 1.27–1.23 (m, 10 H, 5 × CH_2_) and 0.86 (t, *J* = 6.6 Hz, 3H, CH_3_) ppm. Notes; a) more traditional methods of removing the bromine counter ion, such as treatment with Ag(I)O^32^ were not possible as the insolubility of the resulting zwitterion made it impossible to separate the AgBr precipitate from the product; b) zwitterion 3a is insoluble in H_2_O, MeOH and only sparingly soluble in DMSO and H_2_O at pH = 1.

### 3-Carboxy-1-heptylcarbamoylmethyl-pyridinium bromide (26)

Bromoacetyl bromide (6.0 mmol) was reacted with *n*-heptylamine under general protocol A to afford **14** as a waxy solid (1.3 g, 92%). ^1^H NMR (CDCl_3_, 270 MHz) δ 6.62 (brs, 1H, NH), 3.83 (s, 2H, CH_2_CO), 3.24 (m, 2H, CH_2_), 1.49 (m, 2H, CH_2_), 1.23 (m, 8 H, 4 × CH_2_) and 0.83 (m, 3H, CH_3_) ppm; Nicotinic acid (1.62 mmol) and 2-bromo-*N*-heptylacetamide **14** (1.62 mmol) were reacted under general protocol B to afford **26** as a white solid. The compound was further purified by crystallization in MeOH/acetone, giving pure **26** as colourless crystals (366 mg, 63%); mp: 182–184 °C; ^1^H NMR (DMSO, 270 MHz) δ 9.50 (s, 1H, H_N_-2), 9.17 (d, *J*_6,5_ = 6.1 Hz, 1H, H_N_-6), 8.92 (d, *J*_4,5_ = 8.0 Hz, 1H, H_N_-4), 8.66 (m, 1H, NH), 8.20 (dd, *J*_5,4_ = 8.0 and *J*_5,6_ = 6.1 Hz, 1H, H_N_-5), 5.57 (s, 2H, CH_2_CO), 3.10 (m, 2H, CH_2_N), 1.44 (m, 2H, CH_2_), 1.24 (m, 8 H, 4 × CH_2_) and 0.84 (m, 3H, CH_3_) ppm; ^13^C NMR (DMSO, 68 MHz) δ 164.5, 163.6 131.7 (all C), 148.4, 147.9, 146.3, 128.1 (all CH), 62.1, 31.8, 29.4, 28.9, 26.9, 22.6 (all CH_2_) and 14.5 (CH_3_) ppm; *t*_*R*_ = 5.7 mins (solvent: a gradient of 20–95% MeCN against H_2_O over 25 mins). Anal. Calcd for C_15_H_23_N_2_O_3_Br: C, 50.15; H, 6.45; N, 7.80. Found: C, 50.10; H, 6.39; N, 7.76.

### 3-Carboxy-1-hexylcarbamoylmethyl-pyridinium bromide (27)

2-Bromoacetyl bromide and *n*-hexylamine were reacted under general protocol A. The crude material was purified by column chromatography on silica gel using DCM-hexane (10:1 → 1:0 v/v) to afford **15** (60%) as a colourless oil. ^1^H NMR (CDCl_3_, 270 MHz) δ 6.84 (brs, 1H, NH), 3.80 (s, 2H, CH_2_CO), 3.20 (m, 2H, CH_2_), 1.50 (m, 2H, CH_2_), 1.23 (m, 6 H, 3 × CH_2_), 0.82 (m, 3H, CH_3_) ppm; Nicotinic acid (1.62 mmol) and 2-bromo-*N*-hexylacetamide **15** (1.62 mmol) were reacted under general protocol B to afford **27** as a white solid. Further purification by crystallization in MeOH/acetone afforded pure **27** as colourless crystals (319 mg, 57%); mp: 177–179 °C; ^1^H NMR (D_2_O, 270 MHz) δ 9.30 (s, 1H, H_N_-2), 9.04 (d, *J*_6,5_ = 8.1 Hz, 1H, H_N_-6), 8.92 (d, *J*_4,5_ = 6.2 Hz, 1H, H_N_-4), 8.20 (dd, *J*_5,4_ = 8.1 and *J*_5,6_ = 6.2 Hz, 1H, H_N_-5), 5.49 (s, 2H, CH_2_CO), 3.22 (m, 2H, CH_2_N), 1.49 (m, 2H, CH_2_), 1.24 (m, 6 H, 3 × CH_2_) and 0.80 (m, 3H, CH_3_) ppm; ^13^C NMR (D_2_O, 68 MHz) δ 164.5, 163.6, 132.8 (all C), 148.2, 147.1, 146.8, 128.2 (all CH), 62.1, 40.1, 30.7, 28.2, 25.8, 22.0 (all CH_2_) and 13.4 (CH_3_) ppm; *t*_*R*_ = 4.3 mins (solvent: a gradient of 20–95% MeCN against H_2_O over 25 mins). Anal. Calcd for C_14_H_21_N_2_O_3_Br: C, 48.71; H, 6.13; N, 8.11. Found: C, 48.60; H, 6.12; N, 8.07.

### 3-Carboxy-1-[(3-methoxy-benzylcarbamoyl)-methyl]-pyridinium bromide (28)

2-Bromoacetyl bromide and 3-methoxy-benzylamine were reacted under general protocol A. The crude product was purified by flash chromatography, eluted with DCM-hexane 10:1 v/v to afford **16** as a yellow solid (2.25 g, 72%); mp: 79–80 °C; ^1^H NMR (CDCl_3_, 270 MHz) δ 7.24–6.83 (m, 4H, ArH), 4.40 (s, 2H, CH_2_), 3.86 (s, 2H, CH_2_CO) and 3.77 (s, 3H, CH_3_) ppm; ^13^C NMR (CDCl_3_, 100 MHz) δ 165.8, 159.9, 139.1 (all C), 130.0, 120.0, 113.5, 113.2 (all CH), 55.6, 29.4 (both CH_2_) and 44.4 (CH_3_) ppm; *m/z* (FAB^+^) 257.9 [(M + H)^+^, 100%], 259.9 [(M + H)^+^, 95%], 178.0 [(M-Br)^+^, 71%]; HRMS (FAB^+^) calcd. for C_10_H_13_^79^BrNO_2_^+^ 258.0130 [M+H]^+^, found 258.0136; calcd. for C_10_H_13_^81^BrNO_2_^+^ 260.0109 [M+H]^+^, found 260.0119. Nicotinic acid (1.62 mmol) and 2-bromo(3-methoxy-benzyl) acetamide **16** (1.62 mmol) were reacted under general protocol B to afford **28** as a white solid (568 mg, 92%); mp: 194–196 °C; ^1^H NMR (D_2_O, 270 MHz) δ 9.33 (s, 1H, H_N_-2), 9.07 (d, *J*_6,5_ = 7.8 Hz, 1H, H_N_-6), 8.96 (d, *J*_4,5_ = 6.2 Hz, 1H, H_N_-4), 8.24 (dd, *J*_5,6_ = 7.8 and *J*_5,4_ = 6.2 Hz, 1H, H_N_-5), 7.37 (m, 1H, ArH-2), 6.98 (m, 3H, ArH-4, H-5 and H-6), 5.62 (s, 2H, CH_2_CO), 4.47 (s, 2H, CH_2_N) and 3.84 (s, 3H, CH_3_) ppm; ^13^C NMR (D_2_O, 100 MHz) δ 164.9, 163.6, 159.8, 140.5, 130.9 (all C), 149.8, 148.0, 146.5, 130.1, 128.3, 120.1, 113.7, 112.9 (all CH), 62.2, 43.1 (both CH_2_) and 55.7 (CH_3_) ppm; *m/z* (FAB^+^) 301.0 [(M)^+^, 100%]; HRMS (FAB^+^) calcd. for C_16_H_17_N_2_O_4_^+^ 301.1188 [M]^+^, found 301.1200.

### 3-Bromo-5-carboxy-1-decylcarbamoylmethyl-pyridinium bromide (29)

5-Bromo-nicotinic acid (1.62 mmol) and 2-bromo-*N*-decylacetamide (1.62 mmol) **12** were reacted under general protocol B to afford **29** as a white solid (631 mg, 81%); mp: 118–120 °C; ^1^H NMR (DMSO, 270 MHz) δ 9.58 (s, 1H, H_N_-2), 9.53 (s, 1H, H_N_-6), 9.19 (s, 1H, H_N_-4), 8.56 (brs, 1H, NH), 5.45 (s, 2H, CH_2_CO), 3.09 (m, 2H, CH_2_N), 1.42–1.23 (m, 16 H, 8 × CH_2_) and 0.83 (m, 3H, CH_3_) ppm; ^13^C NMR (DMSO, 100 MHz) δ 164.0, 162.7, 146.9, 131.8 (all C), 150.6, 148.6, 121.8 (all CH), 62.2, 34.7, 31.8, 31.2, 29.5, 29.5, 29.3, 29.2, 26.8, 22.6 (all CH_2_) and 14.5 (CH_3_) ppm; HRMS (FAB^+^) calcd. for C_18_H_28_^79^BrN_2_O_3_^+^ 399.1283 [M]^+^, found 399.1290; Calcd. for [M]^+^ C_18_H_28_^81^BrN_2_O_3_^+^ 401.1263 found 401.1274; *t*_*R*_ = 7.4 mins (solvent: a gradient of 35–95% MeCN against H_2_O over 25 mins).

### 3-Carboxy-1-(2-(octylamino)-2-oxoethyl)-5-phenylpyridinium bromide (30)

1) H_2_SO_4_ (2 mL) was added dropwise into a solution of 5-bromo-nicotinic acid (500 mg, 2.5 mmol) in MeOH (12 mL) and the resulting solution was refluxed for 16 h. Saturated aqueous NaHCO_3_ (40 mL) was added slowly and the resulting mixture was diluted with DCM (100 mL). The organic layer was separated, dried over MgSO_4_ and the solvents removed by evaporation to afford the 5-bromo-nicotinic acid methyl ester in quantitative yield.

2) 5-Bromo-nicotinic acid methyl ester (400 mg, 1.87 mmol) and phenyl boronic acid (452 mg, 3.7 mmol) were suspended in toluene (4 mL). To the suspension was added MeOH (1 mL) and an aqueous solution of Na_2_CO_3_ (2 M, 2 mL). The resulting mixture was bubbled with argon for 40 min and Pd(PPh_3_)_4_ (35 mg, 0.03 mmol) was added under argon atmosphere. The reaction mixture was heated at reflux for 4.5 h, then filtered through Celite, diluted with DCM (150 mL), washed with H_2_O (50 mL) and dried over MgSO_4_. The solvent was removed *in vacuo* and the residue was purified by column chromatography on silica gel (DCM: EtOAc 10:1 v/v) giving 5-phenyl nicotinic acid methyl ester (192 mg, 48%). ^1^H NMR δ 9.17 (d, *J*_2,4_ = 1.9 Hz, 1H), 8.98 (d, *J*_6,4_ = 2.2 Hz, 1H) 8.47 (m, 1H), 7.59 (m, 2H), 7.48 (m, 3H) and 3.96 (s, 3H) ppm.

3) To a suspension of KOH (230 mg, 4.1 mmol) in H_2_O (4.6 mL) and MeCN (18 mL) was added 5-phenyl nicotinic acid methyl ester (150 mg, 0.70 mmol). The reaction mixture was heated at reflux for 2 h and the solvent was removed *in vacuo*. The residue was dissolved in MilliQ water (20 mL) and to the clear solution 4 M HCl was added, adjusting the pH to 4. The resulting precipitate was filtered and dried giving 5-phenyl nicotinic acid as a white solid (115 mg, 83%). ^1^H NMR δ 9.12 (s, 1H), 9.07 (s, 1H), 8.46 (s, 1H), 7.81 (m, 2H) and 7.50 (m, 3H) ppm.

4) 5-Phenyl nicotinic acid (58 mg, 0.29 mmol) and **13** (73 mg, 0.29 mmol) were reacted under the general method B giving the title compound as a yellow oil (73 mg, 56%). ^1^H NMR (CD_3_OD, 270 MHz) δ 9.52 (s, 1H, H_N_-2), 9.44 (s, 1H, H_N_-4), 9.25 (s, 1H, H_N_-6), 8.60 (brs, 1H, NH), 7.87 (m, 2H, 2 × ArH), 7.59 (m, 3H, 3 × ArH), 5.67 (s, 2H, CH_2_CO), 3.30 (m, 2H, CH_2_), 1.58 (m, 2H, CH_2_), 1.30 (m, 10 H, 5 × CH_2_), 0.91 (m, 3H, CH_3_) ppm; HRMS (ES^+^) calcd for C_22_H_29_N_2_O_3_^+^ 369.2178 [M]^+^, found 369.2164; *t*_*R*_ = 6.4 mins (solvent: a gradient of 35–95% MeCN against H_2_O over 25 mins).

### 3-Carbamoyl-1-(2-(octylamino)-2-oxoethyl)pyridinium bromide (31)

Nicotinamide (200 mg, 1.64 mmol) and **13** (411 mg, 1.64 mmol) were reacted under general procedure B. Crystallization in MeOH give the title compound as a white solid (547 mg, 90%); ^1^H NMR (DMSO, 270 MHz) δ 9.43 (s, 1H, H_N_-2), 9.09 (d, *J*_6,5_ = 6.3 Hz, 1H, H_N_-6), 9.01 (d, *J*_4,5_ = 8.0 Hz, 1H, H_N_-4), 8.62 (m, 2H, NH_2_), 8.30 (dd, *J*_5,4_ = 8.0 and *J*_5,6_ = 6.3 Hz, 1H, H_N_-5), 8.20 (s, 1H, NH), 5.49 (s, 2H, CH_2_CO), 3.11 (m, 2H, CH_2_), 1.44 (m, 2H, CH_2_), 1.25 (m, 10 H, 5 × CH_2_) and 0.83 (m, 3H, CH_3_) ppm; HRMS (ES^+^) calcd for C_16_H_26_N_3_O_2_^+^ 292.2025 [M]^+^, found 292.2007.

### 1-[(2-Bromo-phenylcarbamoyl)-methyl]-3-methoxycarbonyl-pyridinium (32)

Nicotinic acid methyl ester (1.62 mmol) and 2-bromo-*N*-(2-bromo-phenyl)-acetamide **9** were reacted under the general protocol B giving the desired compound **32** as a white solid (481 mg, 69%); mp: 171–172 °C; ^1^H NMR (DMSO, 270 MHz) δ 10.97 (s, brs, 1H, NH), 9.68 (s, 1H, H_N_-2), 9.26 (d, *J*_6,5_ = 6.2 Hz, 1H, H_N_-6), 9.11 (d, *J*_4,5_ = 8.1 Hz, 1H, H_N_-4), 8.38 (dd, *J*_5,4_ = 8.1 and *J*_5,6_ = 6.2 Hz, 1H, H_N_-5), 7.92 (brs, 1H, ArH), 7.32–7.50 (m, 3H, ArH), 5.78 (s, 2H, CH_2_CO) and 3.99 (s, 3H, CH_3_) ppm; ^13^C NMR (DMSO, 100 MHz) δ 164.1, 162.6, 140.3, 131.7, 129.8 (all C), 150.4, 148.1, 146.6, 128.5, 127.3, 122.3, 122.2, 118.7 (all CH), 63.2 (CH_2_) and 54.4 (CH_3_) ppm; *m/z* (FAB^+^) 349.0, 351.0 [(M+H)^+^, 100%]; HRMS (FAB^+^) calcd. for C_15_H_14_^79^BrN_2_O_3_^+^ 349.0188 [M]^+^, found 349.0181.

### 3-Carboxy-1-(3-(octylamino)-3-oxopropyl)pyridinium bromide (34)

Bromopropionyl chloride (3.4 g, 20 mmol) and *n*-heptylamine (4.6 g, 40 mmol) were reacted under general procedure A giving **17** as a waxy solid. ^1^H NMR (CDCl_3_, 270 MHz) δ 5.69 (brs, 1H, NH), 3.63 (m, 2H, CH_2_), 3.26 (m, 2H, CH_2_), 2.74 (m, 2H, CH_2_), 1.49 (m, 2H, CH_2_), 1.25 (m, 8 H, 4 × CH_2_) and 0.85 (m, 3H, CH_3_) ppm; A solution of nicotinic acid methyl ester (400 mg, 2.92 mmol) and bromopropionylamide **17** (730 mg, 2.92 mmol) in DMF (10 mL) was heated at 60–70 °C for 16 h. DMF was evaporated under reduced pressure and the residue was purified by column chromatography, eluted by 0–8% MeOH against DCM. Compound **33** was given as a yellow oil. ^1^H NMR (CD_3_OD, 270 MHz) δ 9.62 (s, 1H, H_N_-2), 9.29 (d, *J*_6,5_ = 6.0 Hz, 1H, H_N_-6), 9.07 (d, *J*_4,5_ = 8.0 Hz, 1H, H_N_-4), 8.27 (dd, *J*_5,4_ = 8.0 and *J*_5,6_ = 6.0 Hz, 1H, H_N_-5), 5.03 (m, 2H, CH_2_), 4.05 (s, 3H, CH_3_), 3.07 (m, 4H, 2 × CH_2_), 1.42 (m, 2H, CH_2_), 1.25 (m, 8H, 4 × CH_2_) and 0.88 (m, 3H, CH_3_) ppm; **33** was heated at 60 °C for 16 h with HBr (48%, 0.3 mL). Residual HBr was removed *in vacuo* and the title compound was precipitated by addition of acetone in less than 10% yield based on nicotinic acid methyl ester. ^1^H NMR (CD_3_OD, 270 MHz) δ 9.60 (s, 1H, H_N_-2), 9.21 (d, *J*_6,5_ = 6.0 Hz, 1H, H_N_-6), 9.06 (d, *J*_4,5_ = 8.0 MHz, 1H, H_N_-4), 8.22 (dd, *J*_5,4_ = 8.0 and *J*_5,6_ = 6.0 Hz, 1H, H_N_-5), 4.98 (m, 2H, CH_2_, partly overlap with HOD signal), 3.07 (m, 2H, CH_2_), 3.05 (m, 2H, CH_2_), 1.43 (m, 2H, CH_2_), 1.25 (m, 8H, 4 × CH_2_) and 0.88 (m, 3H, CH_3_) ppm; HRMS (ES^+^) calcd for C_16_H_25_N_2_O_3_^+^ 293.1865 [M]^+^, found 293.1839; *t*_*R*_ = 2.4 mins (solvent: a gradient of 35–95% MeCN against H_2_O over 25 mins).

### 3-Carboxy-1-(2-imino-2-(octylamino)ethyl)pyridinium chloride (35)

Chlorocacetonitrile (0.17 mL, 2.68 mmol) was added to a cooled solution of NaOMe (18 mg, 0.33 mmol) in MeOH (5–6 mL) and the turbid solution was stirred at r.t for 1H. Octylamine hydrochloride (550 mg, 3.33 mmol) was added and the resulting clear solution was left at rt for 2 h. Removal of the solvent *in vacuo* gave a dark coloured oil which was then dissolved in DMF (5 mL). Nicotinic acid (200 mg, 1.62 mmol) was added to the solution and the resulting suspension was heated at 60–70 °C for 16 h. DMF was evaporated under reduced pressure and the residue was washed with acetone. Analytical sample was obtained by crystallization from MeOH/acetone (128 mg, 22%); mp: 234–236 °C; ^1^H NMR (D_2_O, 400 MHz) δ 9.38 (s, 1H, H_N_-2), 9.02 (m, 2H, H_N_-4 and H_N_-6), 8.20 (dd, *J*_5,6_ = 7.0 and *J*_5,4_ = 7.4 Hz, 1H, H_N_-5), 5.73 (s, 2H, CH_2_CO), 3.24 (m, 2H, CH_2_), 1.54 (m, 2H, CH_2_), 1.13 (m, 10 H, 5 × CH_2_) and 0.70 (m, 3H, CH_3_) ppm; ^13^C NMR (D_2_O, 100 MHz) 164.7, 158.8, 134.3 (all C), 148.2, 147.8, 147.1, 129.4 (all CH), 59.6, 43.1, 31.1, 28.4, 28.3, 26.4, 26.1, 22.1 (all CH_2_) and 13.5 (CH_3_) ppm; HRMS (ES^+^) calcd. for C_16_H_26_N_3_O_2_^+^ 292.2025 [M]^+^, found 292.2008; HPLC: *t*_*R*_ = 6.4 mins (solvent: a gradient of 20–95% MeCN against H_2_O over 25 mins).

### 3-Carbamoyl-1-(2-methyleneoctyl)pyridinium iodide (37)

1) To a solution of 2-hexylacrolein^37,38^ (10 mmol) in EtOH (15 mL) was added NaBH_4_ (11 mmol). The reaction was stirred at rt for 2 h and was quenched by addition of ice. It was extracted with hexane (2 × 20 mL) and DCM (2 × 20 mL) and the organic layers were combined, dried and evaporated. Purification by column chromatography (DCM-hexane 10:1 → 1:0 v/v) gave **36** as a colourless liquid (47%). NMR data agreed with that reported^37^. To a solution of **36** (300 mg, 2.11 mmol), PPh_3_ (820 mg, 3.13 mmol) and imidazole (217 mg, 3.19 mmol) in dry THF (22 mL) was added I_2_ (795 mg, 3.13 mmol) in one portion at 0 °C under argon. The resulting mixture was stirred at rt for 2 h and the reaction was quenched by addition of Na_2_S_2_O_3_ (10% aq.). The mixture obtained was diluted with ether (100 mL). The organic layer was separated, washed with water and dried over MgSO_4_. The solvent was removed under reduced pressure and the resulting residue was purified by column chromatography (DCM) giving crude iodo-derivative in 86% yield, which was used directly in the next step.

2) Nicotinic acid (74 mg, 0.60 mmol) was added to a solution of the iodo-compound (165 mg, 0.65 mmol) in DMF (4 mL) and the resulting mixture was stirred at 65 °C for 16 h. DMF was evaporated under reduced pressure and the resulting residue was dissolved in small amount of MeOH. Addition of ether to the solution gave the title compound as a yellow oil (125 mg, 55% based on nicotinic acid). ^1^H NMR (CD_3_OD, 270 MHz) δ 9.48 (s, 1H, H_N_-2), 9.19 (d, *J*_6,5_ = 5.9 Hz, 1H, H-6), 9.11 (d, *J*_4,5_ = 8.2 Hz, 1H, H-4), 8.29 (dd, *J*_5,6_ = 8.2 and *J*_5,4_ = 5.9 Hz, 1H, H_N_-5), 5.39 (s, 2H, CH_2_), 5.28 (s, 1H, H-double bond), 5.01 (s, 1H, H-double bond), 2.10 (m, 2H, CH_2_), 1.53 (m, 2H, CH_2_), 1.32 (m, 6 H, 3 × CH_2_) and 0.92 (m, 3H, CH_3_) ppm; ^13^C NMR (CD_3_OD, 100 MHz) 163.8, 133.6 (all C), 148.8, 147.5, 144.6, 129.8, 117.3 (all CH), 67.0, 34.2, 32.7, 29.9, 28.3, 23.6 (all CH_2_) and 14.4 (CH_3_) ppm. HRMS (ES^+^) calcd. for C_15_H_22_NO_2_^+^ 248.1651 [M]^+^, found 248.1637.

### 1-(2-(Heptylamino)-2-oxoethyl)-1,4-dihydropyridine-3-carboxylic acid (38)

A suspension of **26** (200 mg, 0.56 mmol) and NaHCO_3_ (236 mg, 2.81 mmol) in MilliQ water was bubbled under argon in a sonicator for 40 min. Na_2_S_2_O_4_ (292 mg, 1.68 mmol) was added under argon and to the suspension MeOH (15 mL) was added. The resulting clear solution was stirred at rt under argon for 1H during which a white precipitate was produced. CHCl_3_ (15 mL) was added and the mixture was stirred for a further hour. The organic layer was separated and the aqueous phase was extracted with CHCl_3_ (2 × 15 mL). The organic layers were combined, dried over MgSO_4_ and the solvent was removed *in vacuo*, giving the title compound as a yellow solid (120 mg, 77%). The compound was used in the biological assay without further purification. ^1^H NMR (CDCl_3_, 270 MHz) δ 7.03 (s, 1H, H_N_-2), 5.96 (m, 1H, NH), 5.64 (d, *J*_6,5_ = 8.5 Hz, 1H, H_N_-6), 4.91 (m, 1H, H_N_-5), 3.76 (s, 2H, CH_2_CO), 3.27 (m, 2H, CH_2_), 3.09 (m, 2H, H_N_-4), 1.52 (m, 2H, CH_2_), 1.27 (m, 8 H, 4 × CH_2_) and 0.86 (m, 3H, CH_3_) ppm; HRMS (ES^+^) calcd for C_15_H_24_N_2_NaO_3_^+^ 303.1679 [M + Na]^+^, found 303.1674.

### 2-Methyl-1-*H*-benzoimidazole-4-carboxylic acid (41)

**41** was synthesized from di-amino compound **39** according to White *et al*.^39^ in 48% yield. ^1^H NMR (DMSO, 270 MHz) δ 8.13 (d, *J* = 7.4 Hz, 1H, ArH), 7.94 (d, *J* = 8.0 Hz, 1H, ArH), 7.62 (dd, *J* = 8.0 and 7.4 Hz, 1H, ArH) and 3.30 (s, 3H, CH_3_) ppm; HRMS calcd for [M + H]^+^ C_9_H_9_N_2_O_2_^+^ (ES^+^) 177.0664, found 177.0652. **42** was synthesized in the same method in less than 10% yield. ^1^H NMR (CD_3_OD, 270 MHz) 8.16 (d, *J* = 7.2 Hz, 1H, ArH), 7.99 (d, *J* = 7.7 Hz, 1H, ArH), 7.66 (m, 1H, ArH), 3.25 (m, 2H, CH_2_), 1.97 (m, 2H, CH_2_) and 1.08 (m, 3H, CH_3_) ppm; HRMS (ES^+^) calcd for C_11_H_13_N_2_O_2_^+^ 205.0977 [M + H]^+^, found 205.0965. **43** was synthesized from **40** using the same method. ^1^H NMR (DMSO, 270 MHz) δ 8.25 (s, 1H, ArH), 8.05 (d, *J* = 7.4 Hz), 7.84 (d, *J* = 7.4 Hz, 1H1H, ArH) and 2.82 (s, 3H, CH_3_) ppm; HRMS (ES^+^) calcd for C_9_H_9_N_2_O_2_^+^ 177.0664 [M + H]^+^, found 177.0664.

## Electronic supplementary material

Supplementary Information

## Data Availability

Crystallographic data for **3** and **3a** have been deposited with the Cambridge Structural Database, deposition numbers CCDC 1553678 and 1829104 respectively. Copies of these data can be obtained free of charge via http://www.ccdc.cam.ac.uk/conts/retrieving.html (or from the Cambridge Crystallographic Data Centre, 12, Union Road, Cambridge, CB2 1EZ, UK; Fax: +44 1223 336033; e-mail: deposit@ccdc.cam.ac.uk). All other data generated during this study are included in this published article and Supplementary Information file.
