# Supplementary material for: Small Molecule Antagonists of NAADP-Induced Ca2+ Release in T-Lymphocytes Suggest Potential Therapeutic Agents for Autoimmune Disease
Source: Sci Rep. 2018 Nov 13;8:16775. doi: 10.1038/s41598-018-34917-3 (PMC6233153; doi:10.1038/s41598-018-34917-3)
Supplement: Supplementary file 1 — Supplementary Information [file 41598_2018_34917_MOESM1_ESM.doc]

**Supporting Information**

**Small-molecule Antagonists of NAADP-Induced Ca^2+^ Release in T-Lymphocytes Suggest Potential Therapeutic Agents for Autoimmune Disease**

Bo Zhang^2^, Joanna M Watt,^1,2^ Chiara Cordiglieri^3^,*[[1]](#footnote-2)§*, Werner Dammermann^4,#^, Mary F. Mahon^5^, Alexander Flügel^3,6^, Andreas H. Guse^4^ and Barry V. L. Potter^1,2*^

^1^Medicinal Chemistry & Drug Discovery, Department of Pharmacology, University of Oxford, Mansfield Road, Oxford OX1 3QT, UK, ^2^Wolfson Laboratory of Medicinal Chemistry, University of Bath, Department of Pharmacy and Pharmacology, Claverton Down, Bath BA2 7AY, UK, ^3^Max-Planck-Institute for Neurobiology, Martinsried, Germany, ^4^The Calcium Signalling Group, Department of Biochemistry and Molecular Cell Biology, University Medical Centre Hamburg-Eppendorf, Martinistrasse 52, D-20246, Hamburg, Germany, ^5^Department of Chemistry, University of Bath, Claverton Down, Bath BA2 7AY, UK, ^6^University Medical Center Göttingen, Institute for Multiple Sclerosis Research, Department of Neuroimmunology, Von-Siebold-Str. 3a, 37075 Göttingen, Germany

**Table of Contents:**

**1. Table of CHN microanalysis data and table of HPLC parameters S2**

**2. HPLC traces of 2 and 3 S3**

**3. Crystallographic data for 3 and** **3a S4**

**4. ^1^H NMR spectra of all new compounds S6**

Table 1: CHN microanalysis of small-molecule NAADP analogues

| Compound | Formula | Calculated | | | Found | | |
| --- | --- | --- | --- | --- | --- | --- | --- |
|  |  | C | H | N | C | H | N |
| **3** | C_16_H_25_N_2_O_3_^+^Br^-^ | 51.48 | 6.75 | 7.50 | 50.60 | 6.76 | 7.36 |
| **19** | C_12_H_17_N_2_O_3_^+^Br^-^ | 45.44 | 5.40 | 8.83 | 44.60 | 5.26 | 8.56 |
| **21** | C_16_H_17_N_2_O_3_^+^Br^-^ | 52.62 | 4.69 | 7.67 | 52.40 | 4.63 | 7.38 |
| **22** | C_14_H_19_N_2_O_3_^+^Br^-^ | 48.99 | 5.58 | 8.16 | 48.90 | 5.46 | 8.04 |
| **25** | C_18_H_29_N_2_O_3_^+^Br^-^ | 53.87 | 7.28 | 6.98 | 54.00 | 7.30 | 6.99 |
| **26** | C_15_H_23_N_2_O_3_^+^Br^-^ | 50.15 | 6.45 | 7.80 | 50.10 | 6.39 | 7.76 |
| **27** | C_14_H_21_N_2_O_3_^+^Br^-^ | 48.71 | 6.13 | 8.11 | 48.60 | 6.12 | 8.07 |

Table 2: HPLC of small-molecule NAADP inhibitors

| Compound | *tR* (min) | Purity (%) | Solvent |
| --- | --- | --- | --- |
| **19** | 4.6 | 100% | 5-50% MeCN against H_2_O over 30 min |
| **20** | 6.6 | 95% | 5-50% MeCN against H_2_O over 30 min |
| **22** | 2.9 | 100% | 20-95% MeCN against H_2_O over 25 min |
| **23** | 4.9 | 98% | 20-95% MeCN against H_2_O over 25 min |
| **2** | 5.3 | 99% | 20-95% MeCN against H_2_O over 25 min |
| **25** | 4.2 | 100% | 35-95% MeCN against H_2_O over 25 min |
| **3** | 7.3 | 100% | 20-95% MeCN against H_2_O over 25 min |
| **26** | 5.7 | 99% | 20-95% MeCN against H_2_O over 25 min |
| **27** | 4.3 | 95% | 20-95% MeCN against H_2_O over 25 min |
| **28** | 3.2 | 100% | 20-95% MeCN against H_2_O over 25 min |
| **29** | 7.4 | 100% | 35-95% MeCN against H_2_O over 25 min |
| **30** | 6.4 | 97% | 35-95% MeCN against H_2_O over 25 min |
| **34** | 2.4 | 100% | 35-95% MeCN against H_2_O over 25 min |
| **35** | 6.4 | 100% | 20-95% MeCN against H_2_O over 25 min |

**HPLC trace of 2**

**HPLC trace of 3**

**Crystallographic data for 3**

| Table 1 Crystal data and structure refinement for h06farm4. | |
| --- | --- |
| Identification code | h06farm4 |
| Empirical formula | C_16_H_24.5_N_2_O_3_Br_0.5_ |
| Formula weight | 332.83 |
| Temperature/K | 150.15 |
| Crystal system | triclinic |
| Space group | P-1 |
| a/Å | 8.7780(1) |
| b/Å | 9.2460(1) |
| c/Å | 21.3480(4) |
| α/° | 91.924(1) |
| β/° | 99.210(1) |
| γ/° | 102.398(1) |
| Volume/Å^3^ | 1666.26(4) |
| Z | 4 |
| ρ_calc_g/cm^3^ | 1.327 |
| μ/mm^‑1^ | 1.281 |
| F(000) | 704.0 |
| Crystal size/mm^3^ | 0.3 × 0.3 × 0.07 |
| Radiation | MoKα (λ = 0.71073) |
| 2Θ range for data collection/° | 7.616 to 54.964 |
| Index ranges | -11 ≤ h ≤ 11, -11 ≤ k ≤ 11, -27 ≤ l ≤ 27 |
| Reflections collected | 25522 |
| Independent reflections | 7559 [R_int_ = 0.0404, R_sigma_ = 0.0419] |
| Data/restraints/parameters | 7559/2/402 |
| Goodness-of-fit on F^2^ | 1.027 |
| Final R indexes [I>=2σ (I)] | R_1_ = 0.0321, wR_2_ = 0.0767 |
| Final R indexes [all data] | R_1_ = 0.0445, wR_2_ = 0.0820 |
| Largest diff. peak/hole / e Å^-3^ | 0.31/-0.67 |

**Crystallographic data for 3a**

| Table 2 Crystal data and structure refinement for s17bvlp1. | |
| --- | --- |
| Identification code | s17bvlp1 |
| Empirical formula | C_16_H_26_N_2_O_4_ |
| Formula weight | 310.39 |
| Temperature/K | 150.00(10) |
| Crystal system | monoclinic |
| Space group | P2_1_/c |
| a/Å | 21.6725(5) |
| b/Å | 10.3908(2) |
| c/Å | 7.22786(17) |
| α/° | 90 |
| β/° | 98.845(2) |
| γ/° | 90 |
| Volume/Å^3^ | 1608.32(7) |
| Z | 4 |
| ρ_calc_g/cm^3^ | 1.282 |
| μ/mm^‑1^ | 0.750 |
| F(000) | 672.0 |
| Crystal size/mm^3^ | 0.127 × 0.103 × 0.046 |
| Radiation | CuKα (λ = 1.54184) |
| 2Θ range for data collection/° | 8.258 to 146.2 |
| Index ranges | -26 ≤ h ≤ 26, -12 ≤ k ≤ 12, -8 ≤ l ≤ 7 |
| Reflections collected | 12941 |
| Independent reflections | 3201 [R_int_ = 0.0352, R_sigma_ = 0.0277] |
| Data/restraints/parameters | 3201/3/209 |
| Goodness-of-fit on F^2^ | 1.040 |
| Final R indexes [I>=2σ (I)] | R_1_ = 0.0413, wR_2_ = 0.1092 |
| Final R indexes [all data] | R_1_ = 0.0502, wR_2_ = 0.1148 |
| Largest diff. peak/hole / e Å^-3^ | 0.42/-0.23 |

**^1^H NMR of novel compounds**


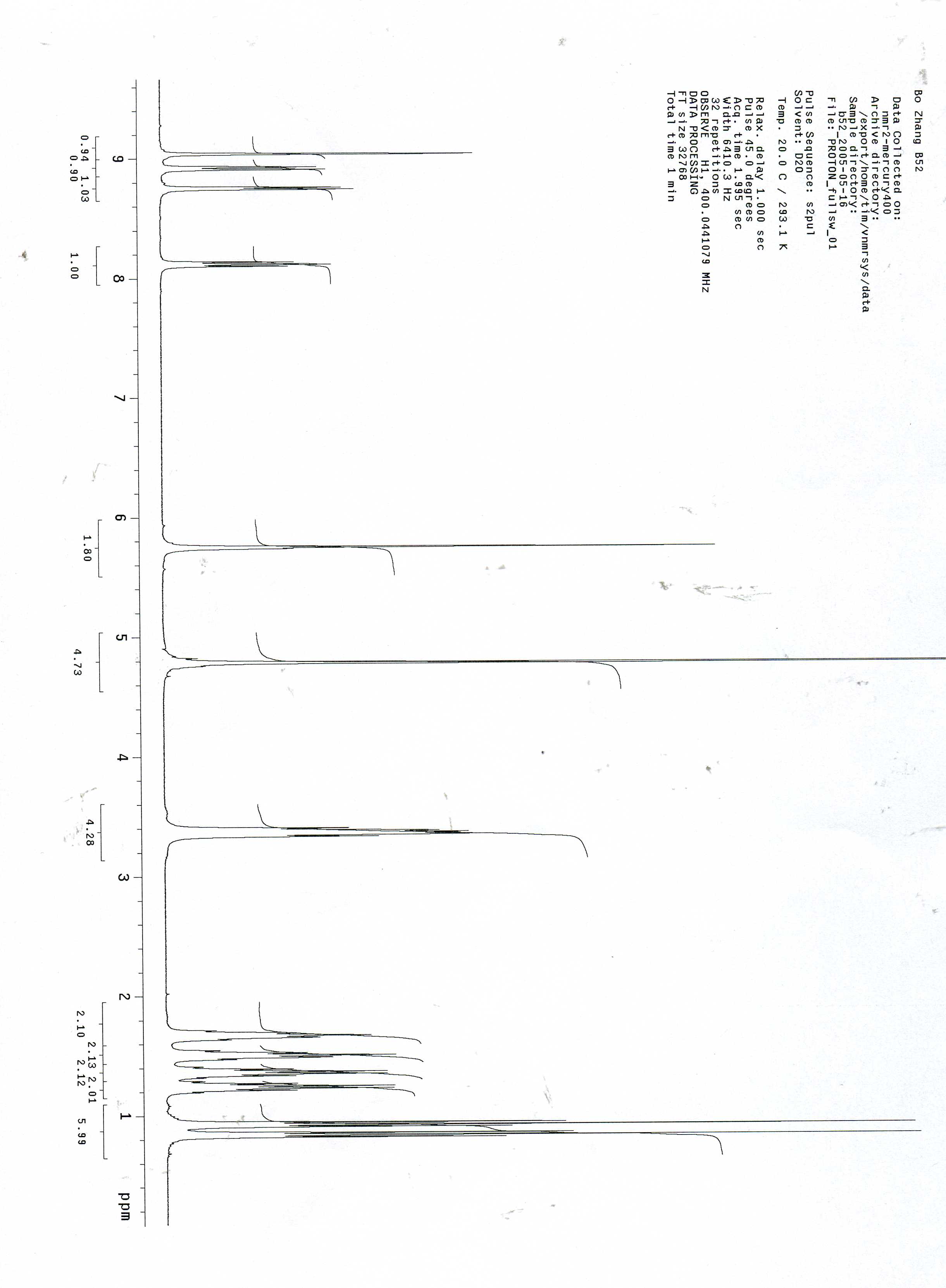


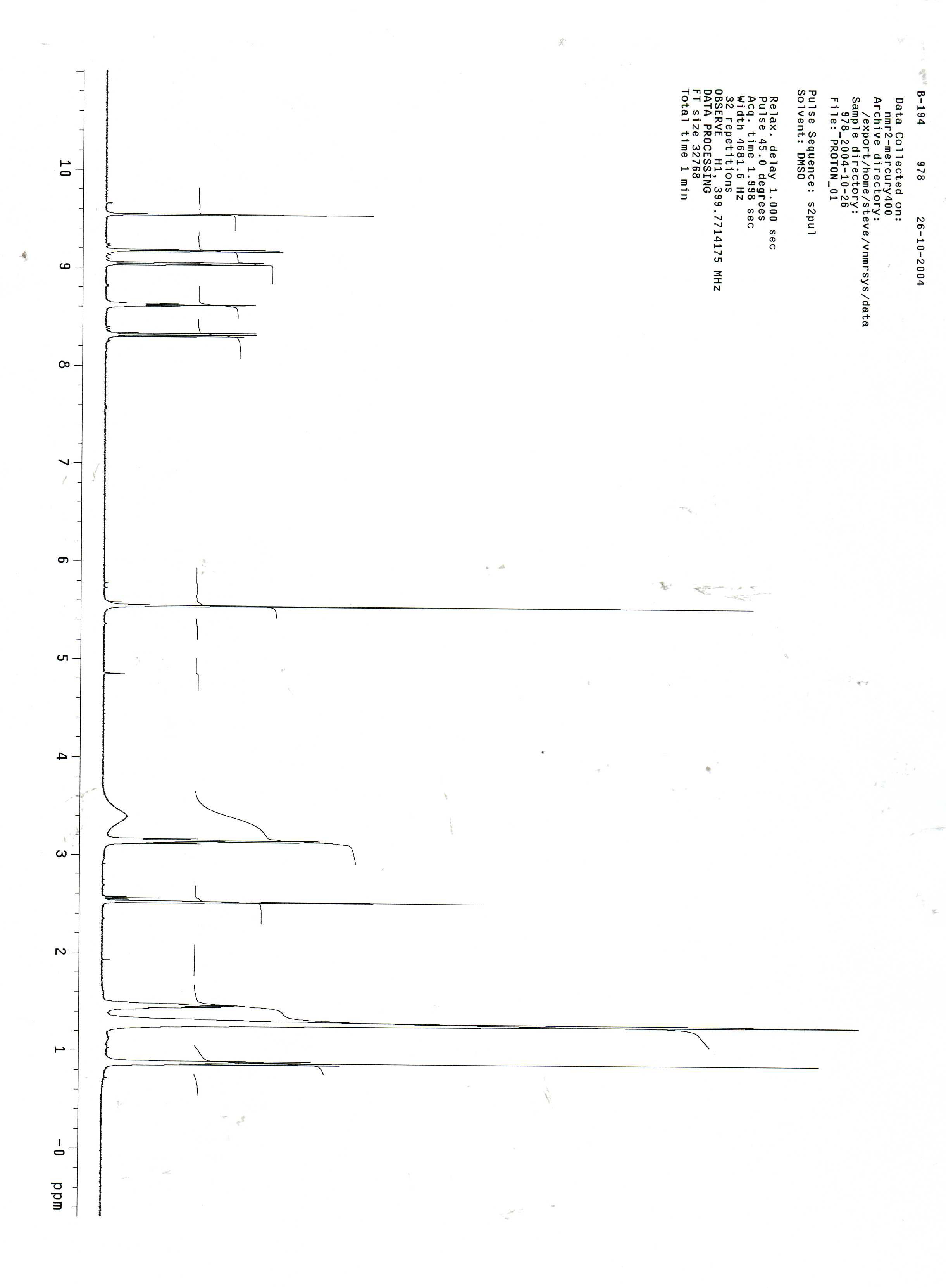


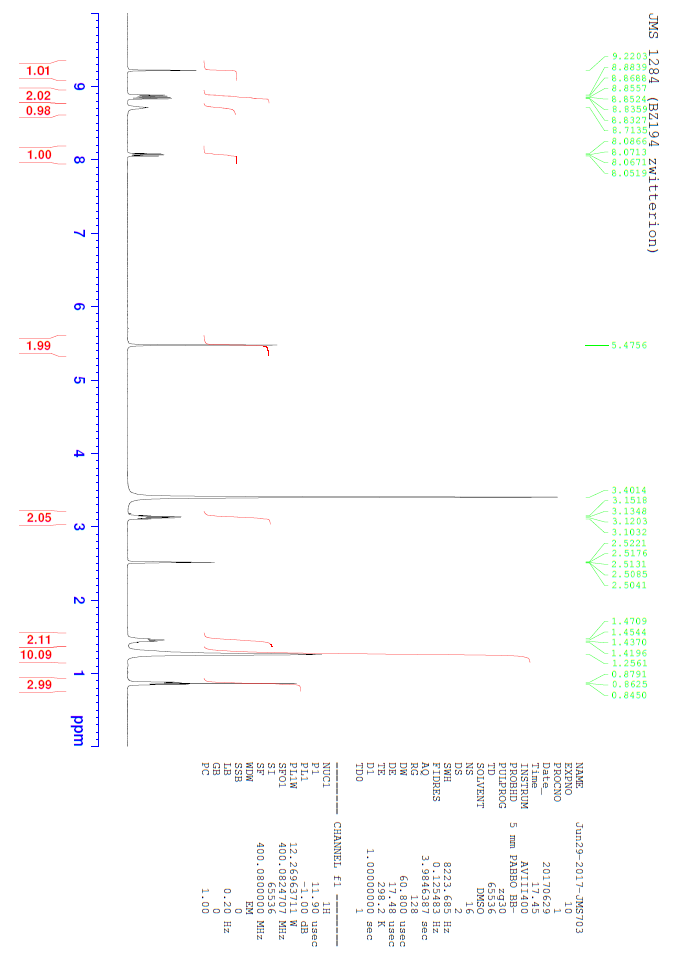


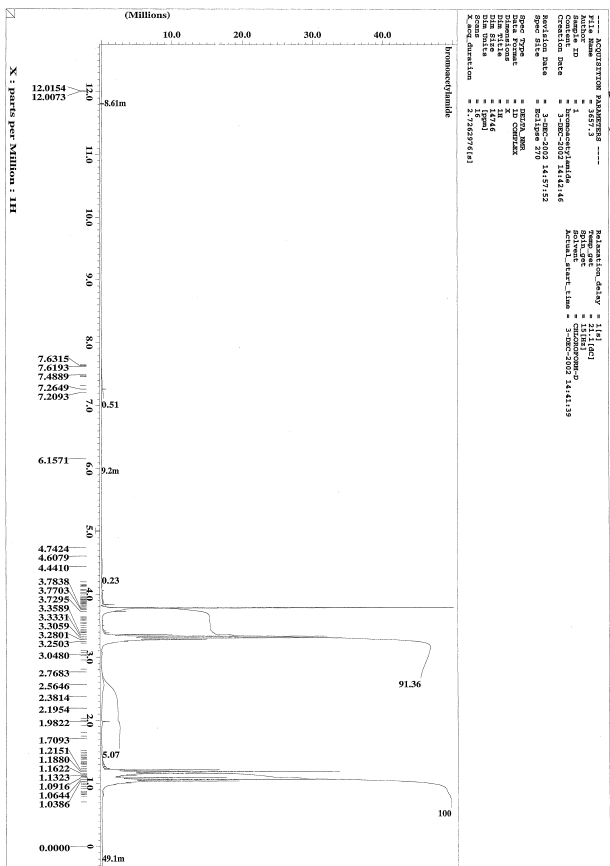


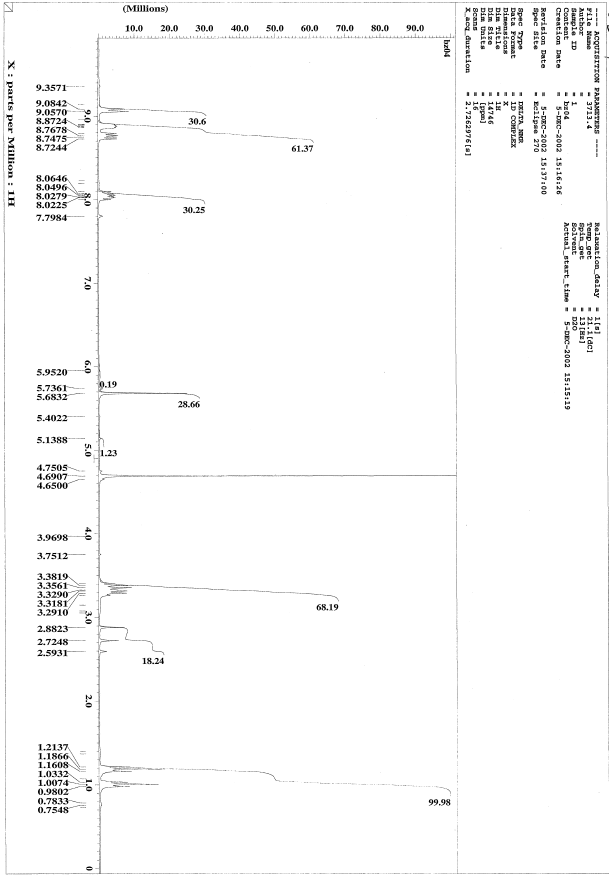


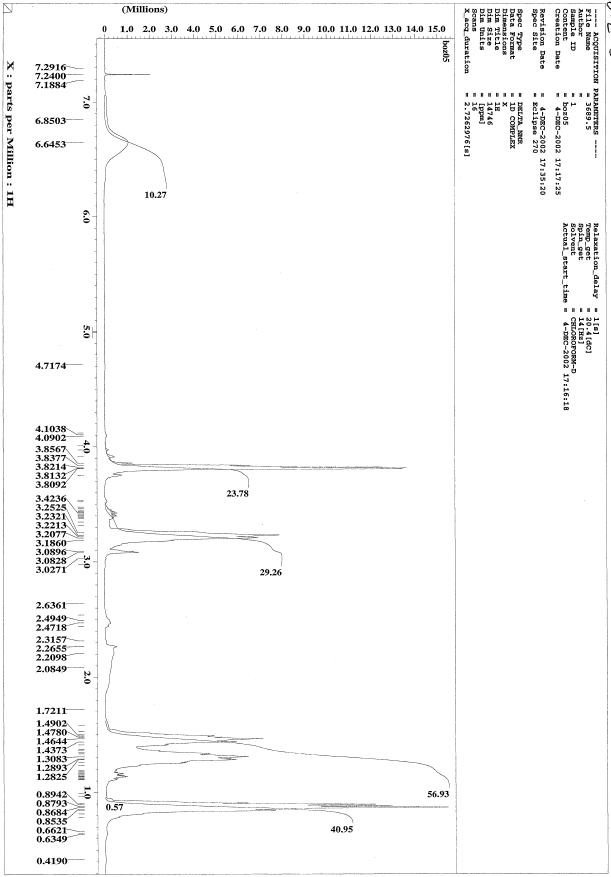


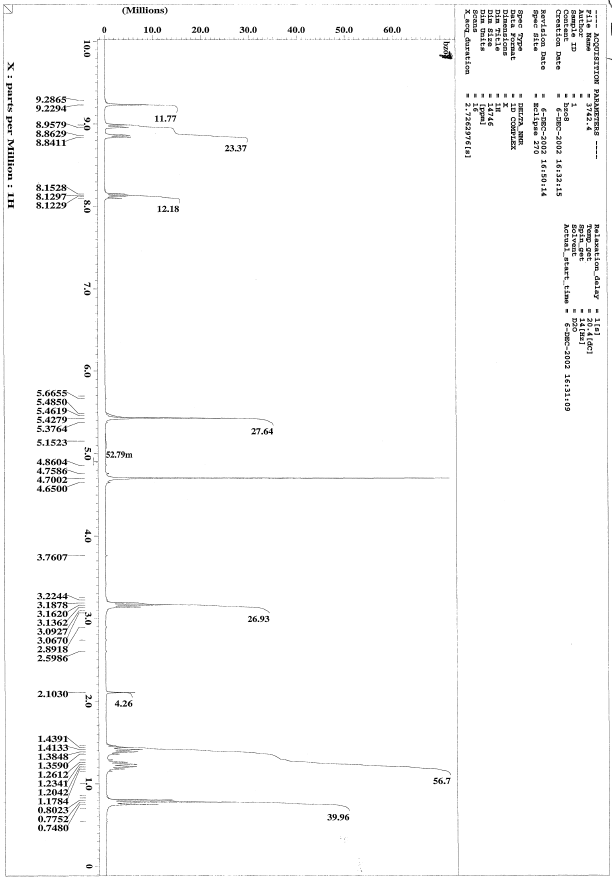


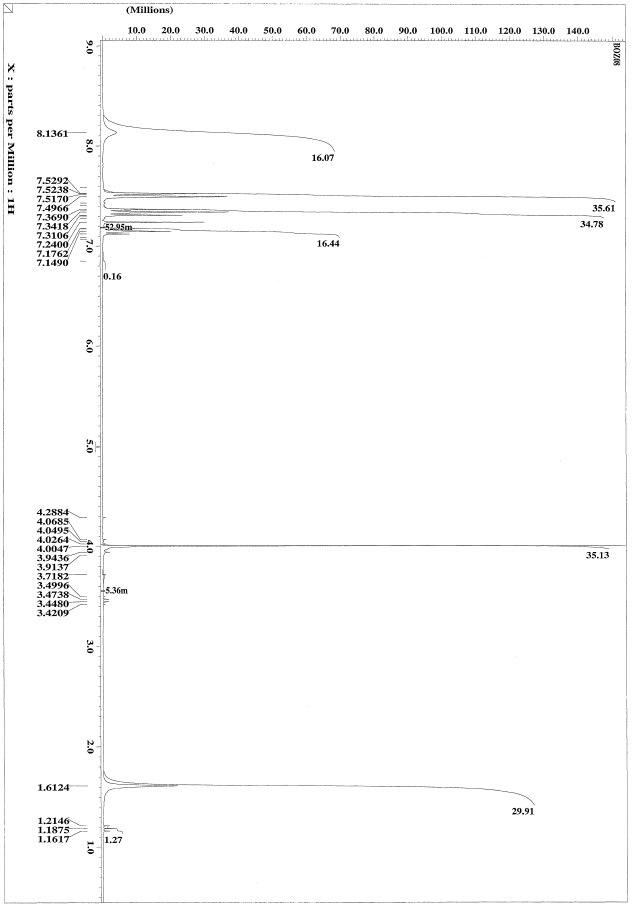


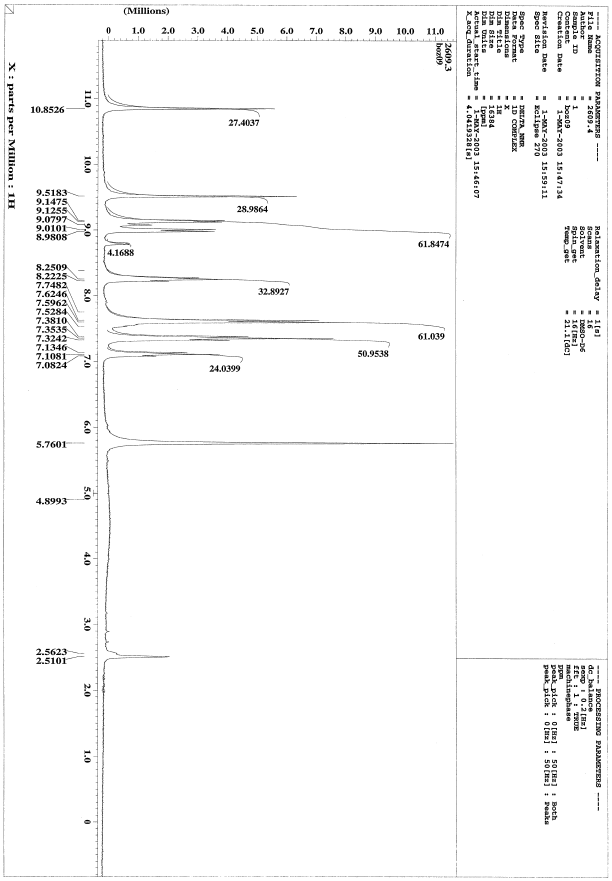


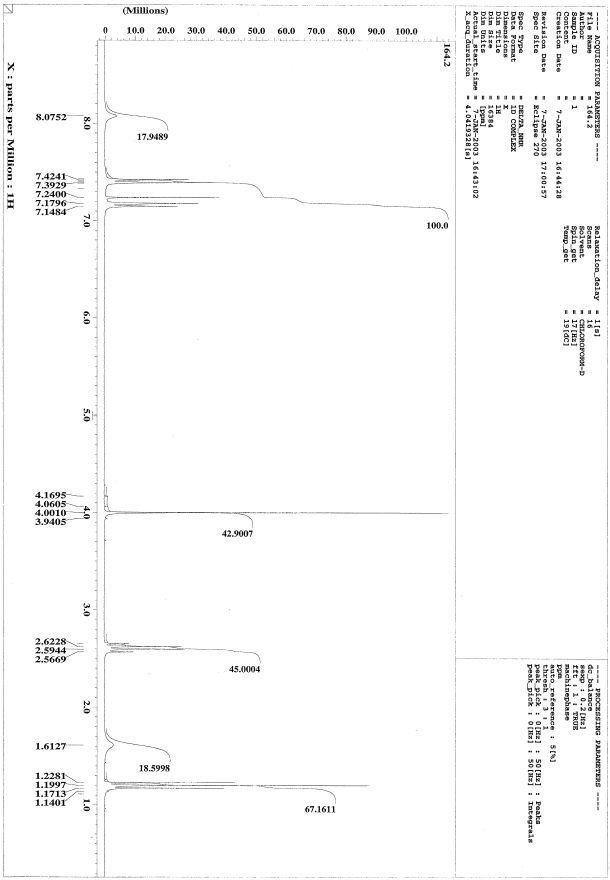


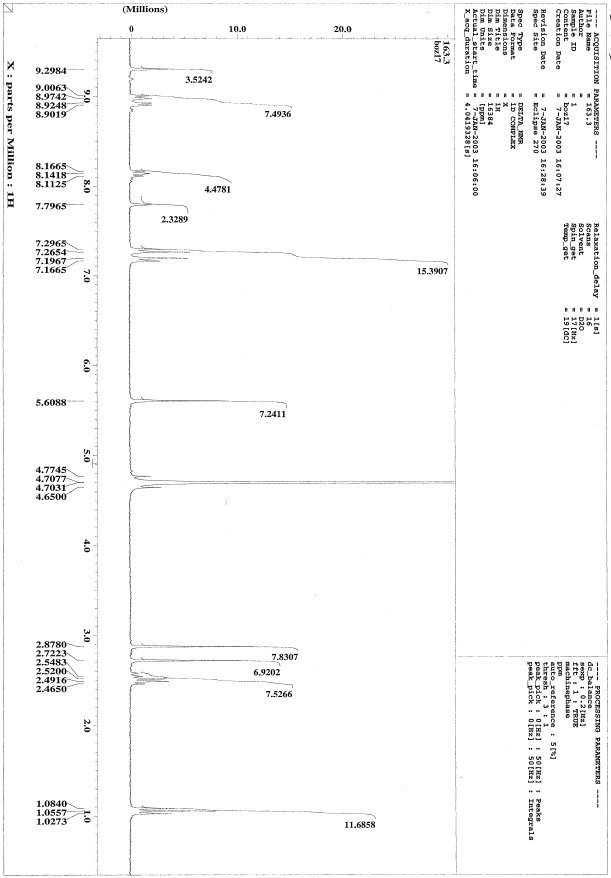


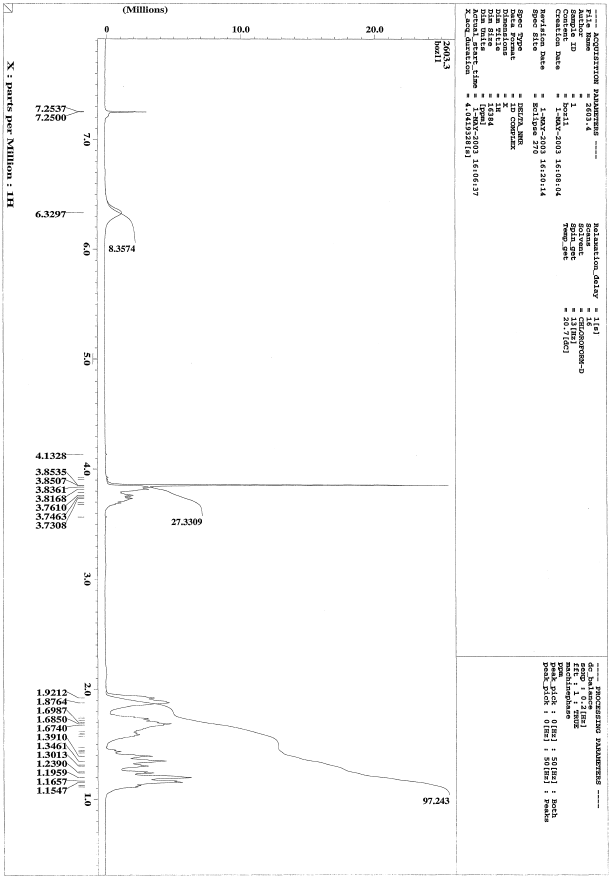


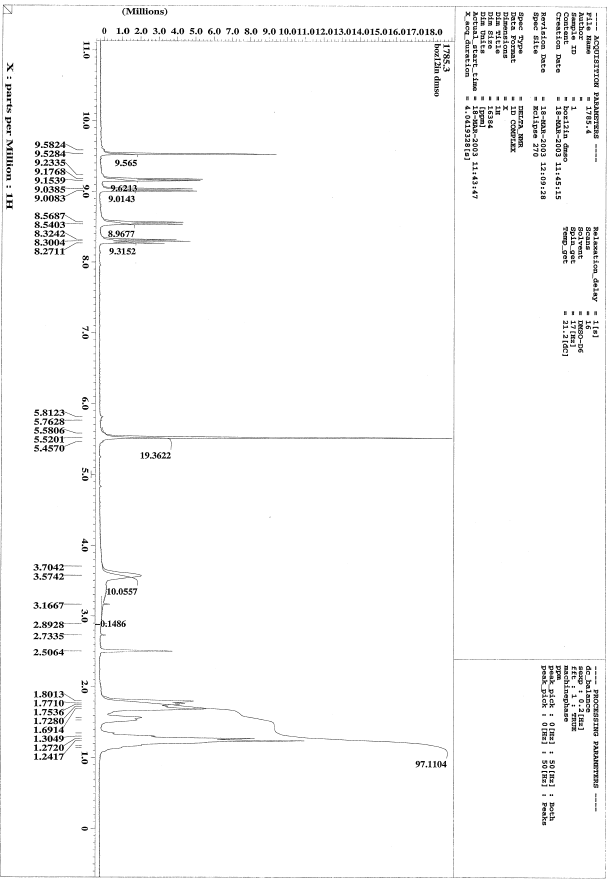


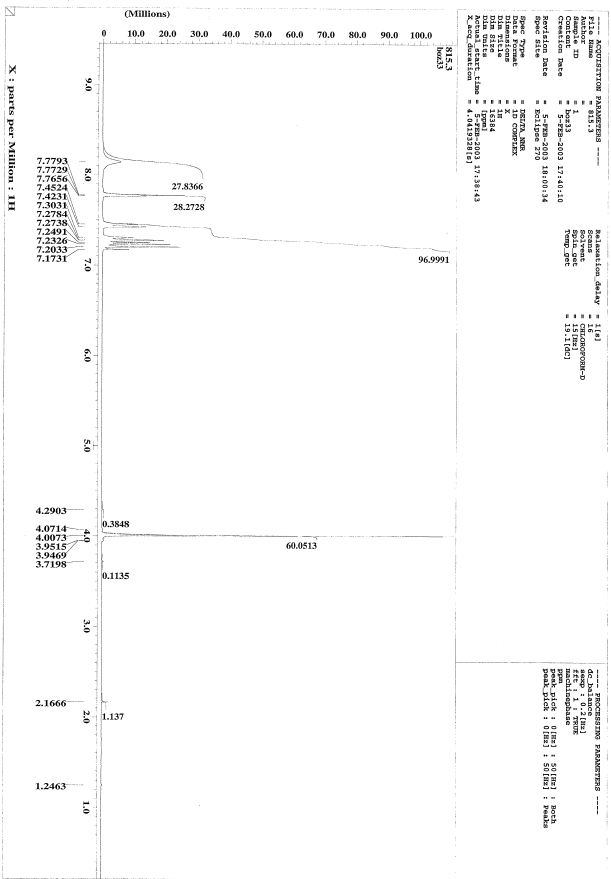


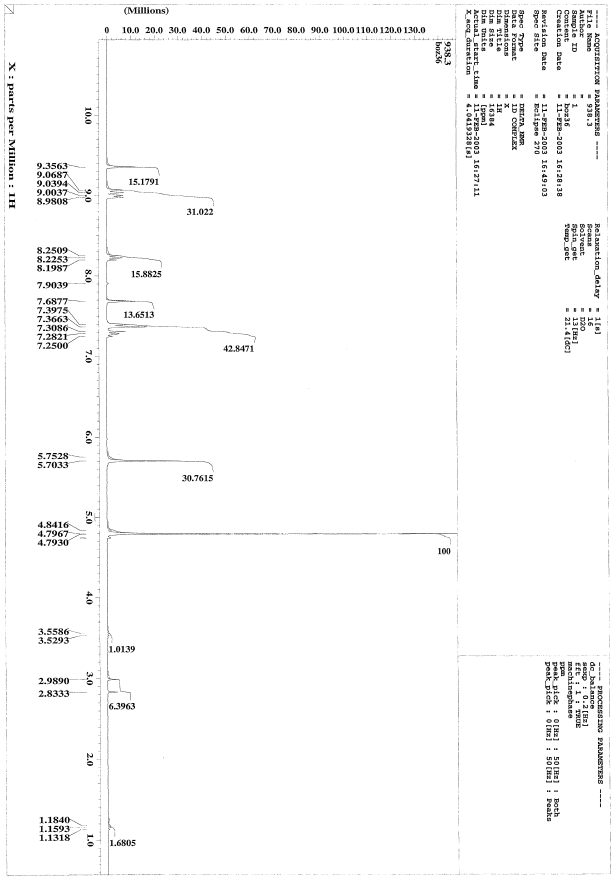


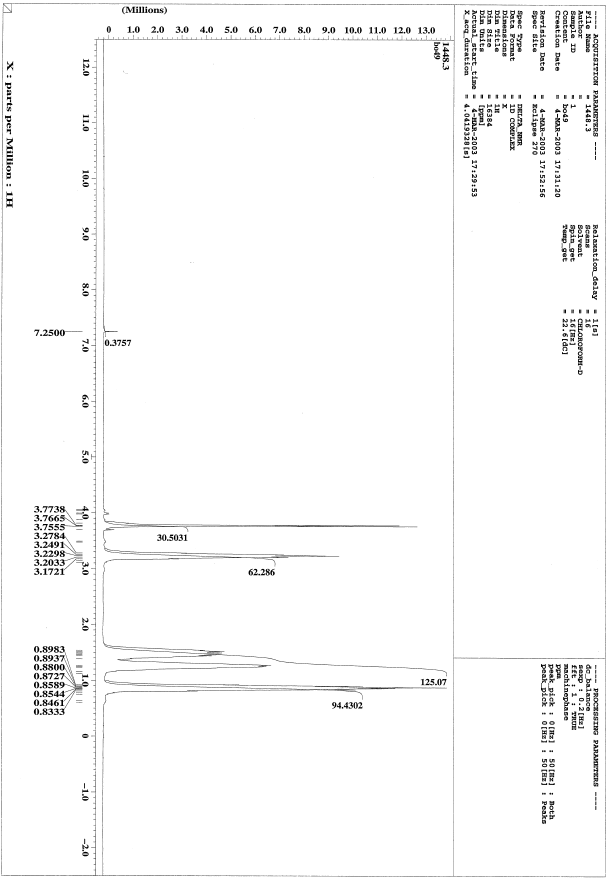


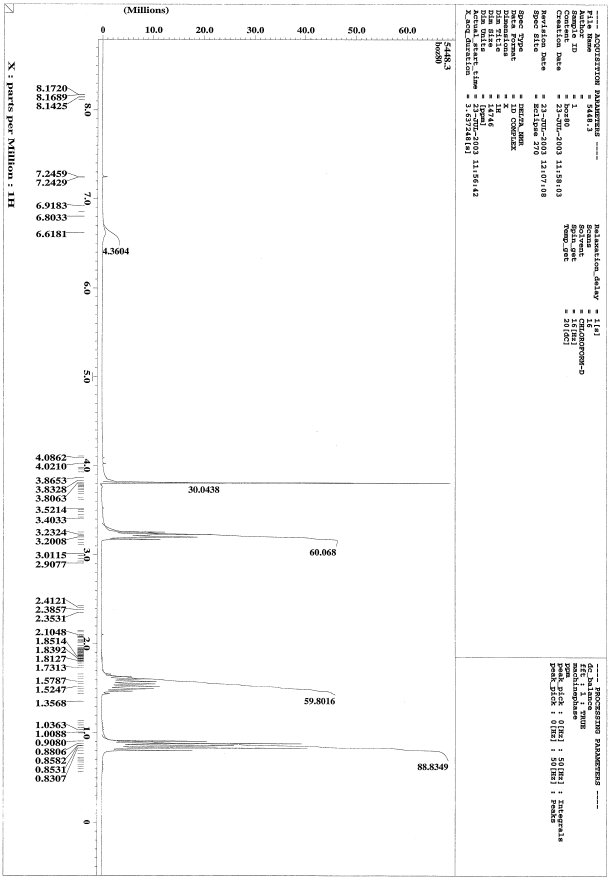


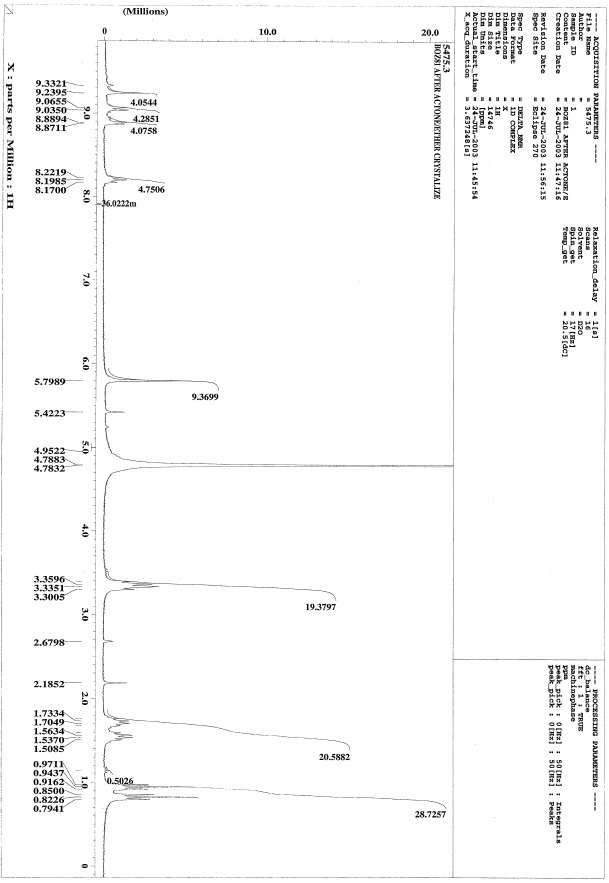


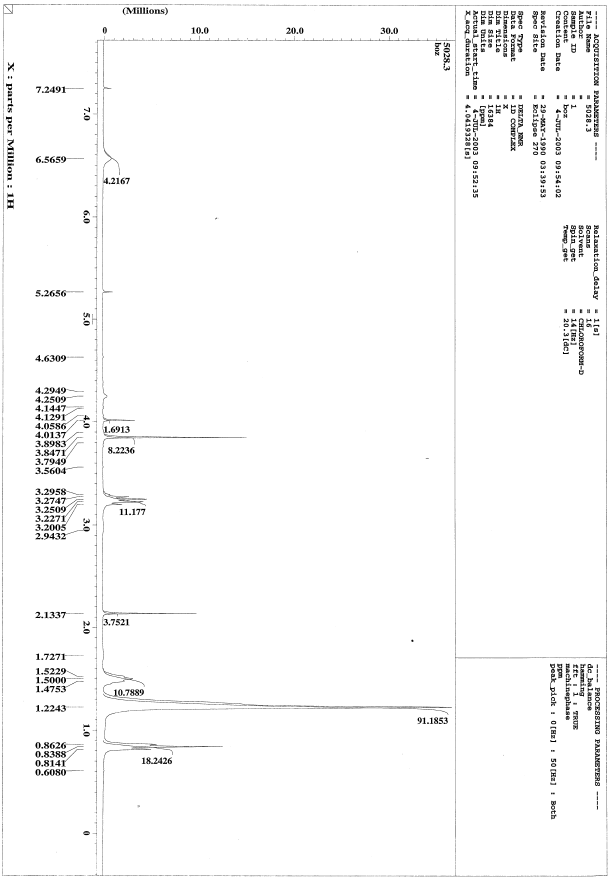


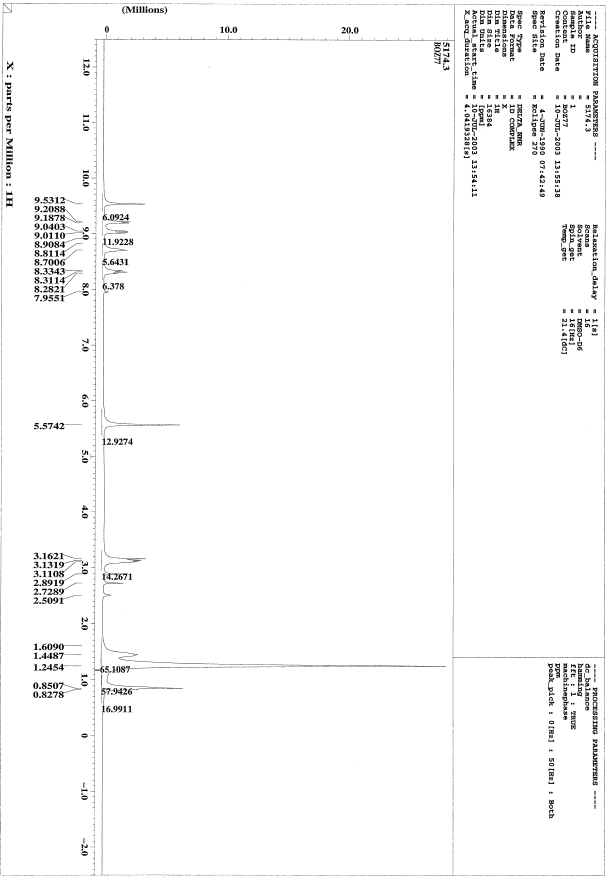


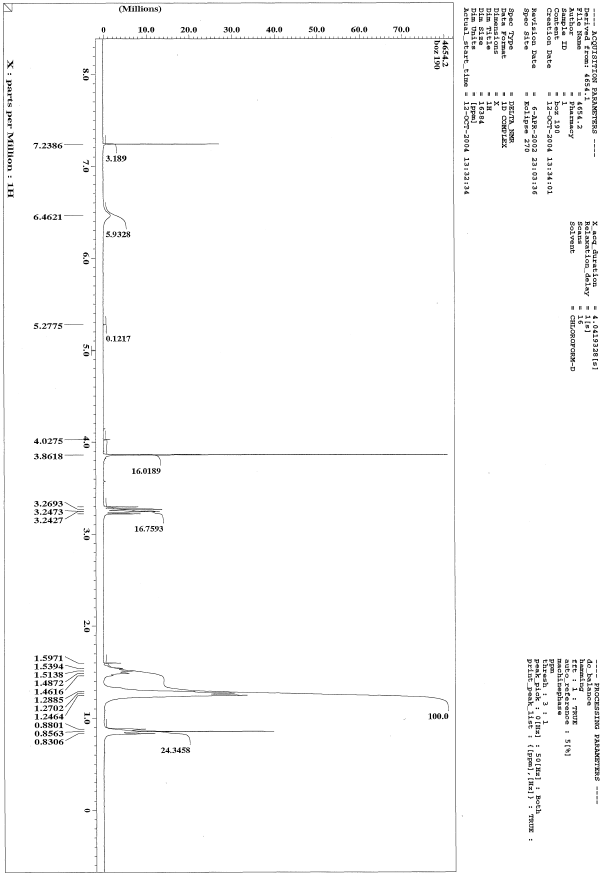


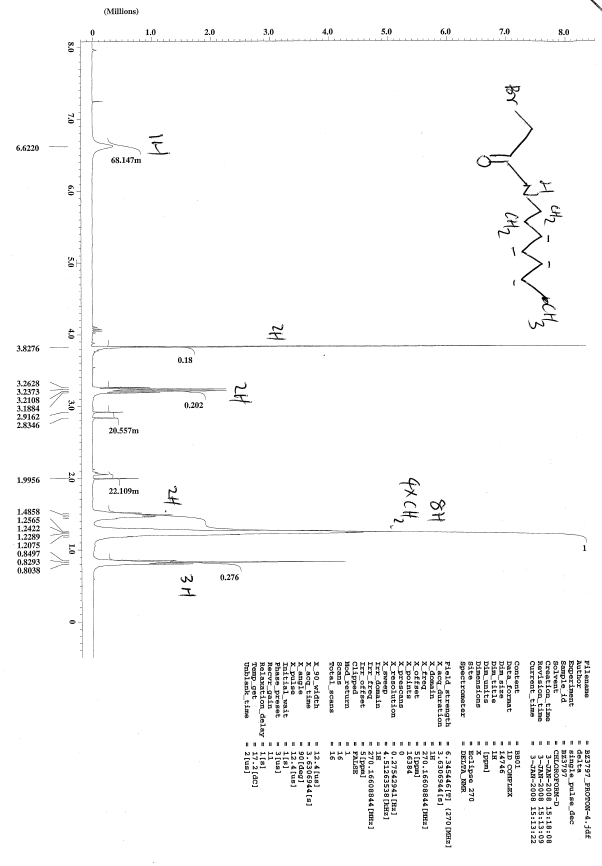


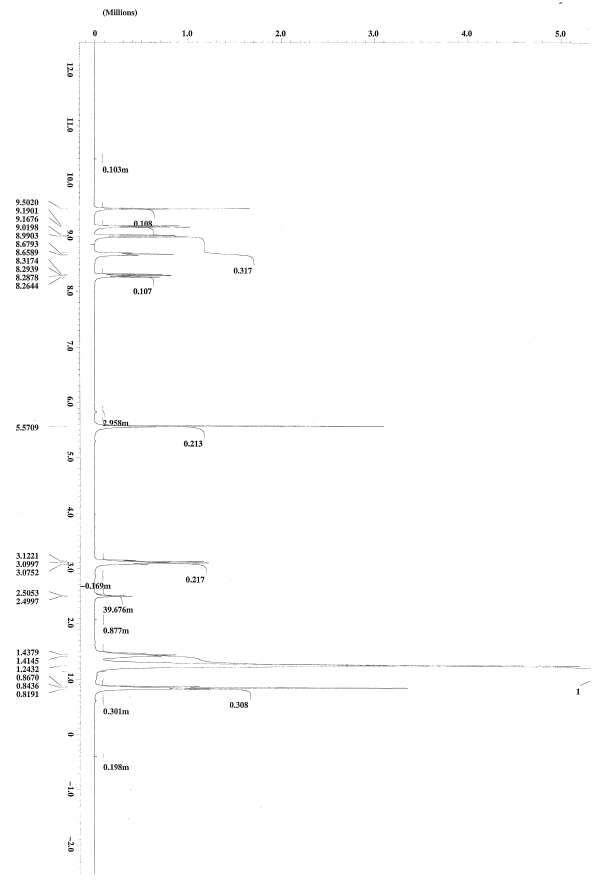


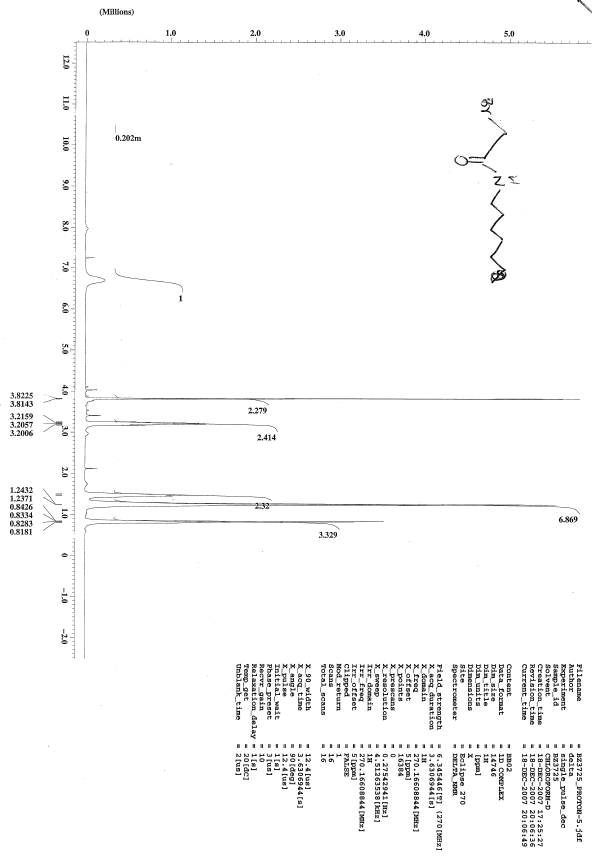


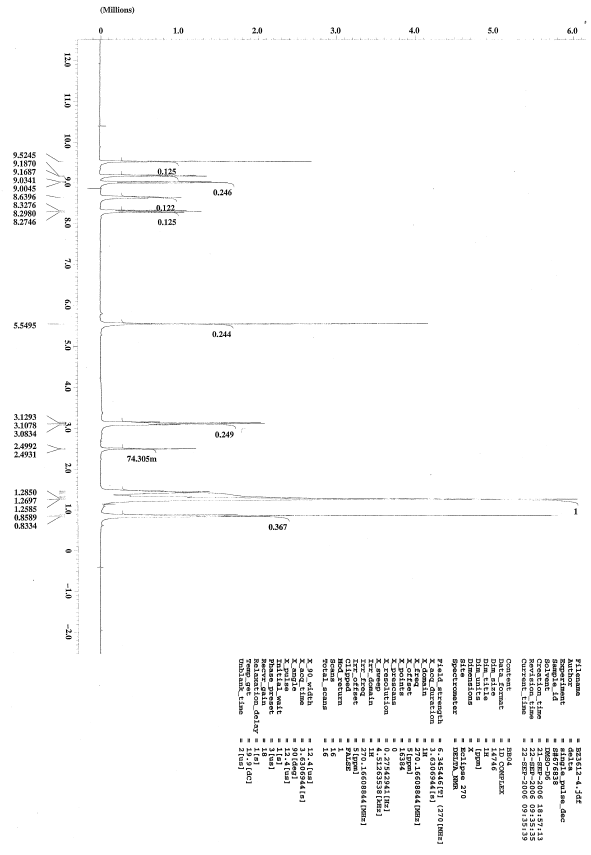


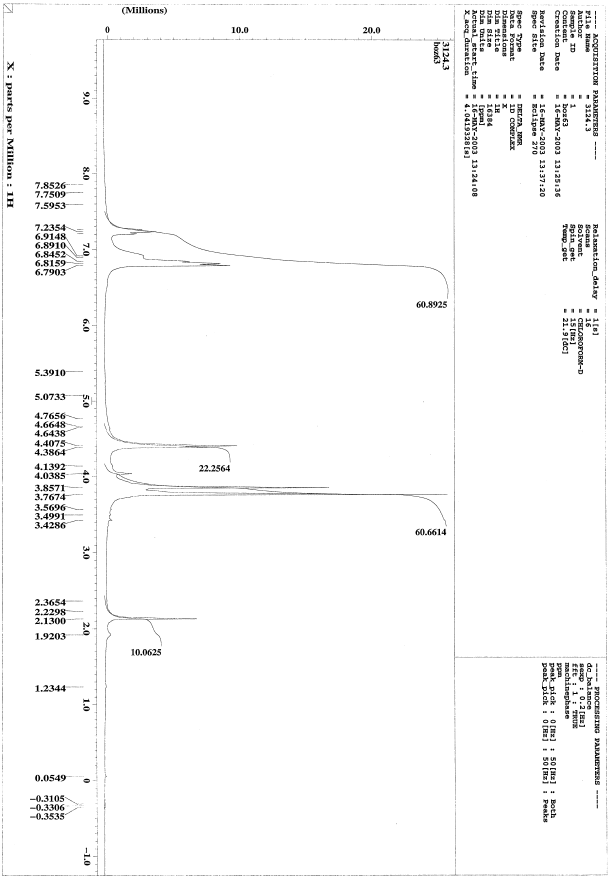


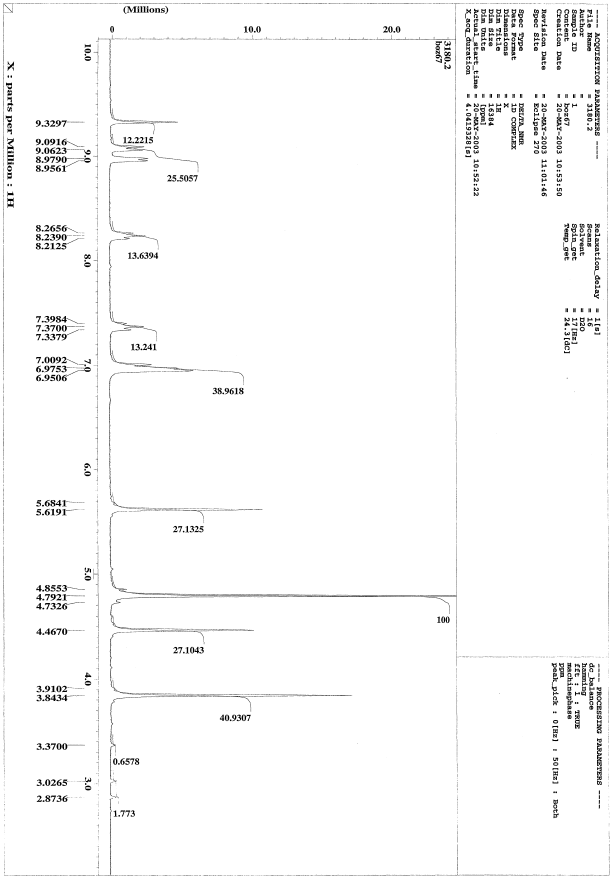


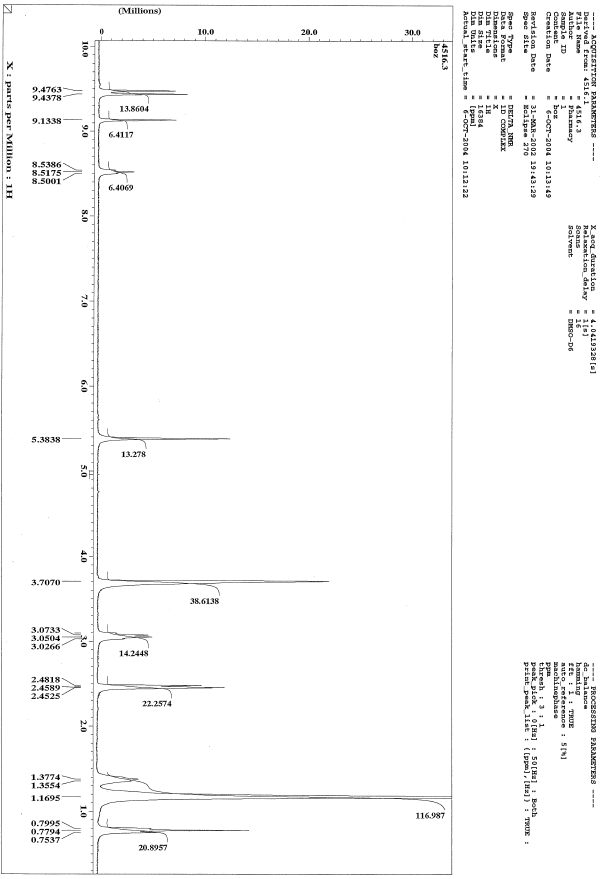


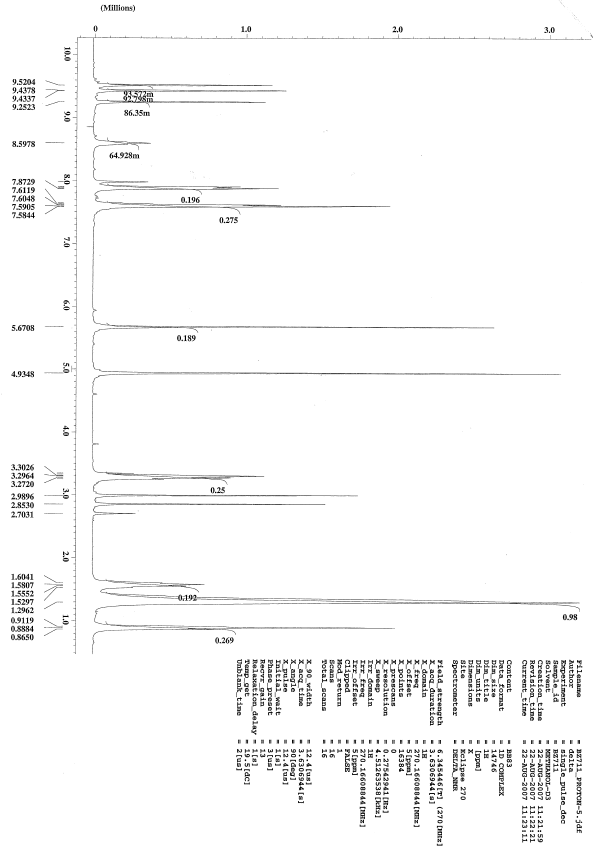


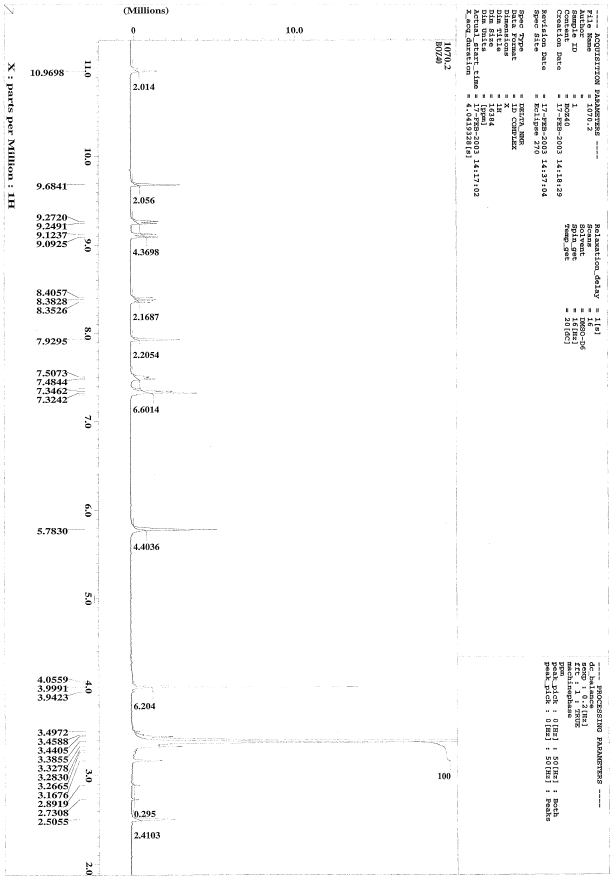


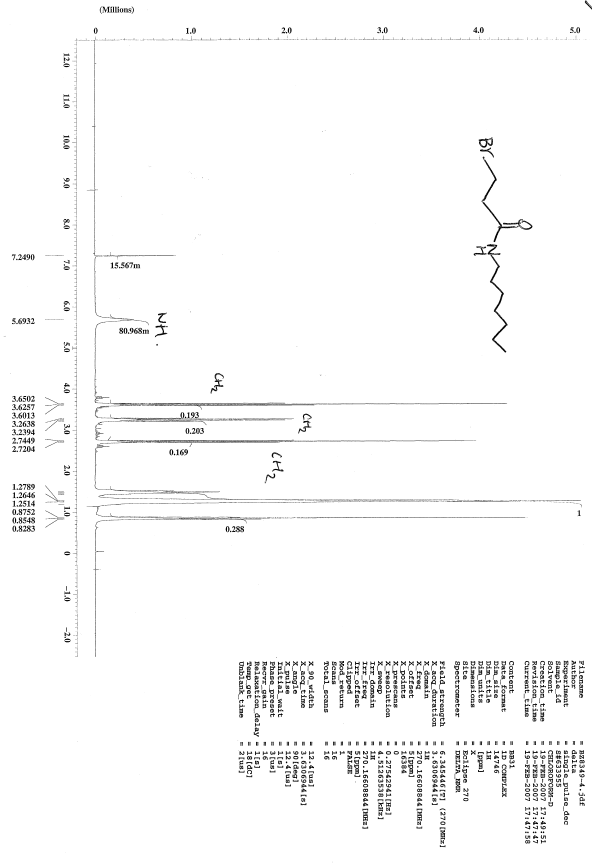


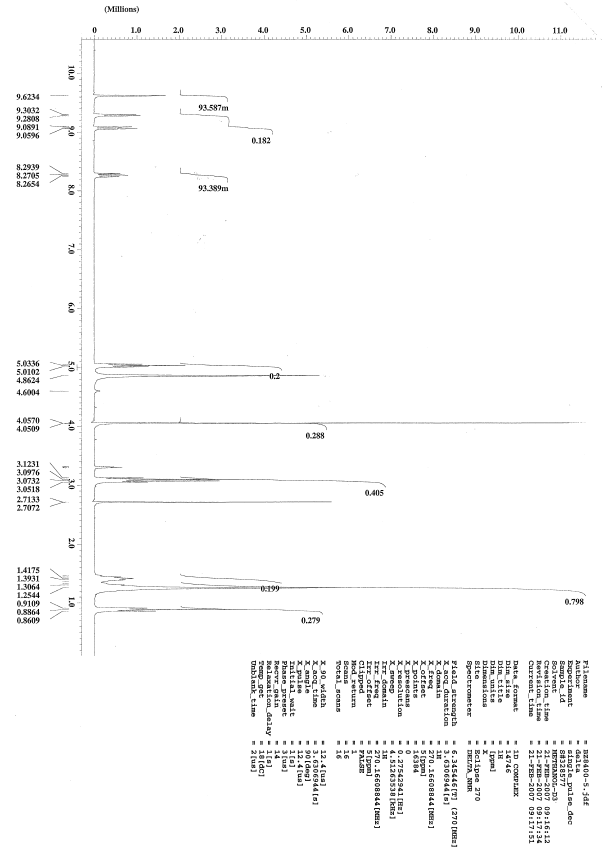


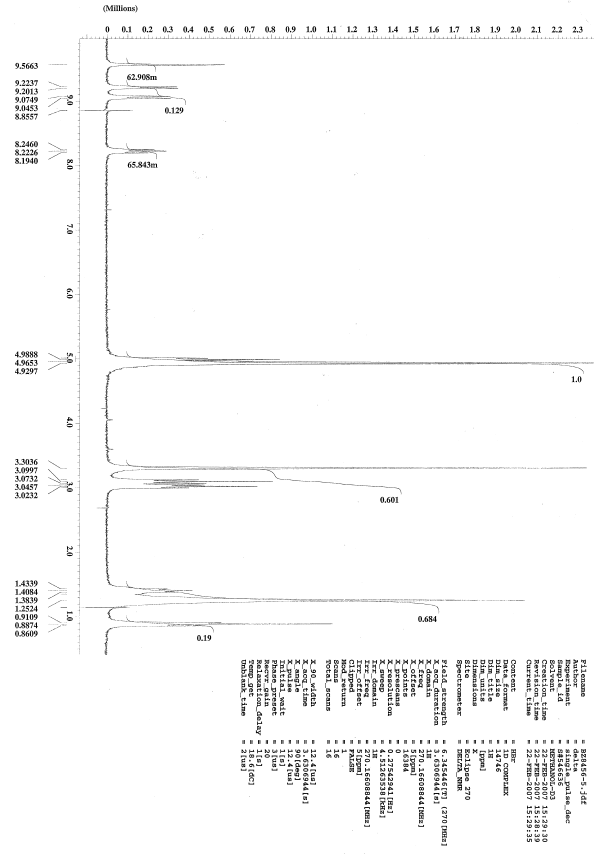


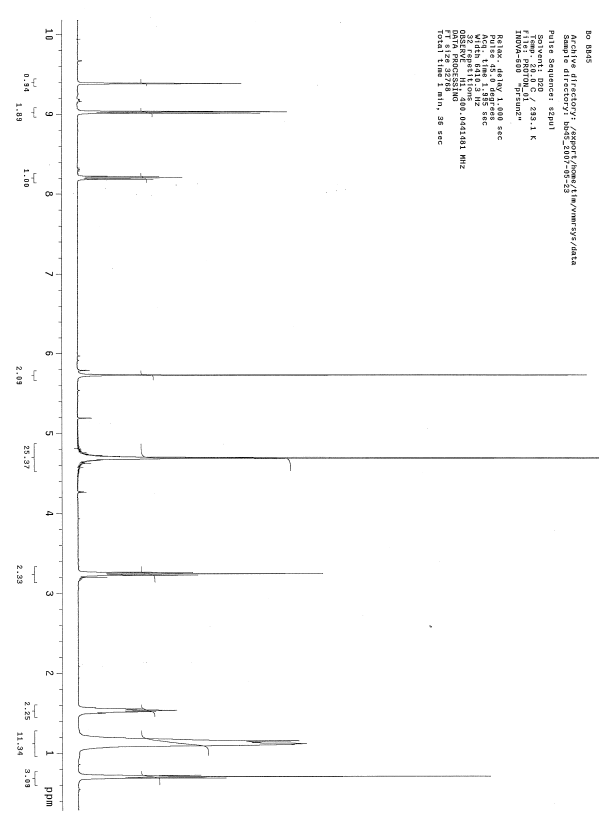


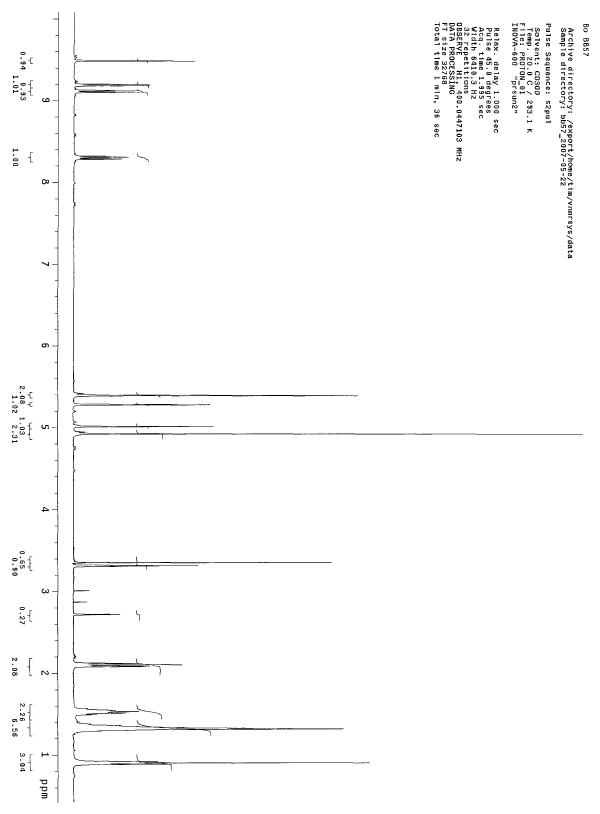


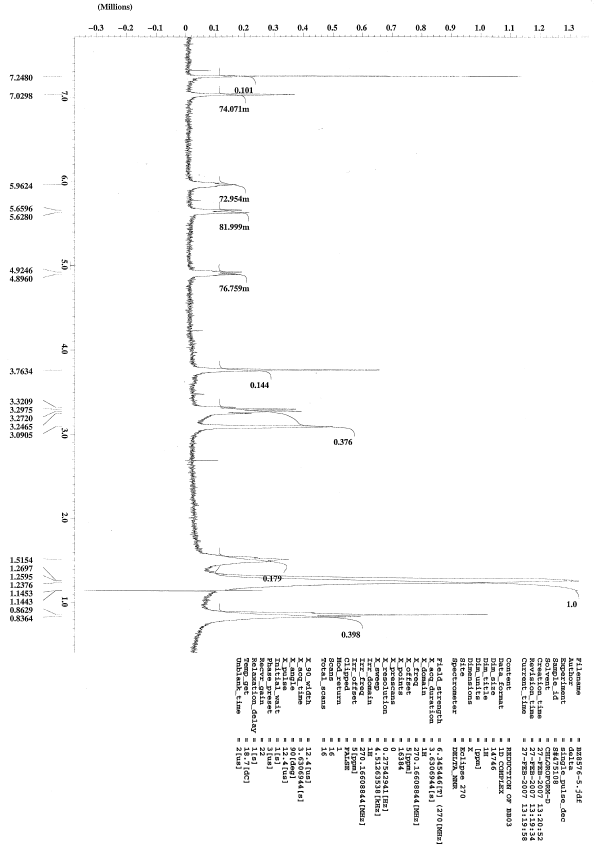


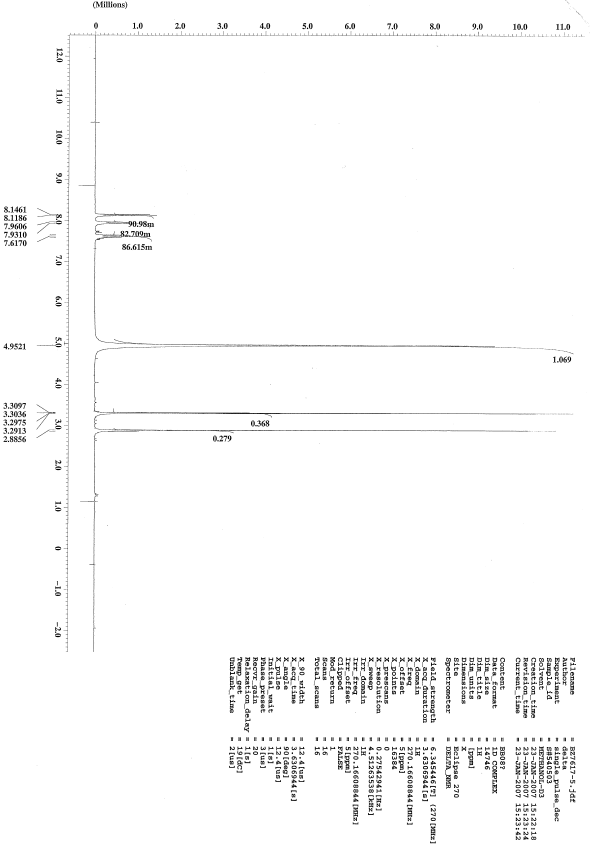


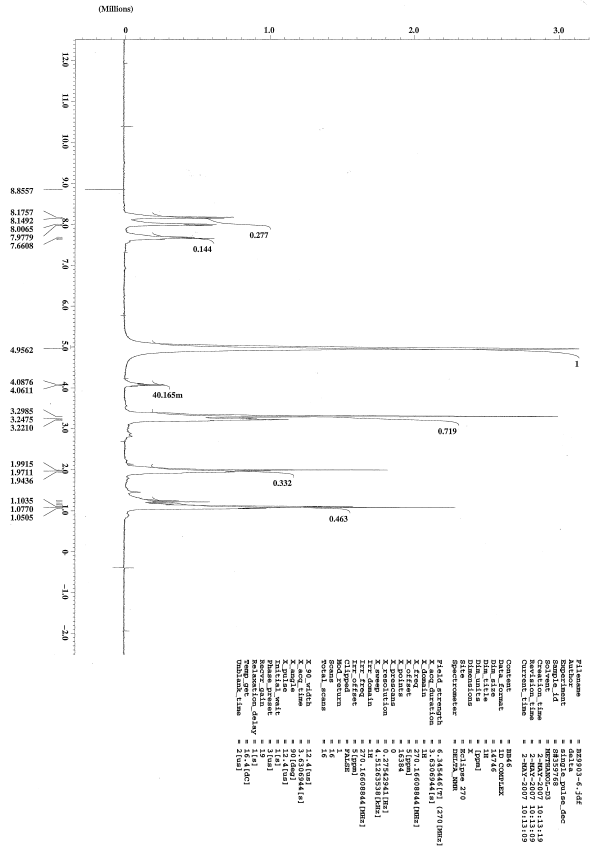


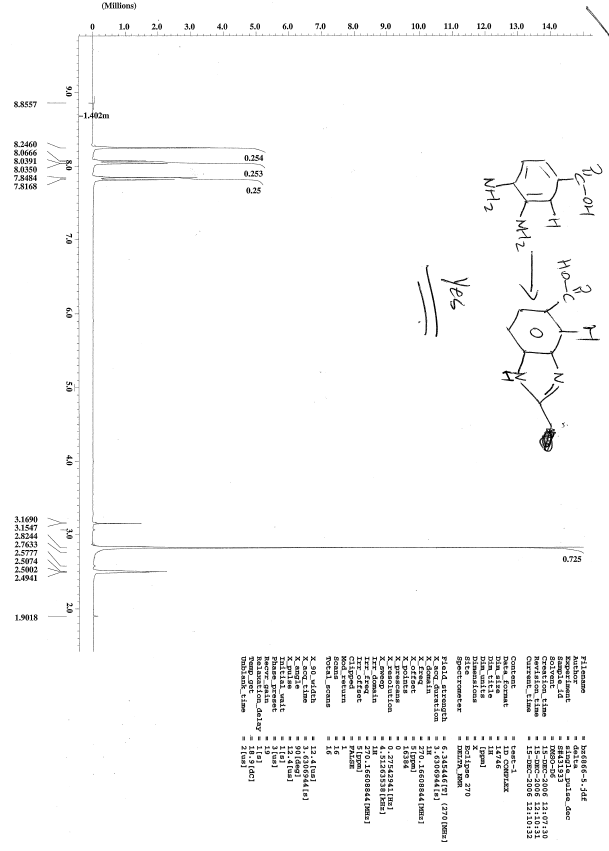


1. ^§^ Present address: Imaging Facility, National Institute for Molecular Genetics (INGM), v. F. Sforza, 35-20122 Milan, Italy

   ^#^Present address: Brandenburg Medical School, University Hospital Brandenburg, Center of Internal Medicine II, Hochstraße 29, 14770 Brandenburg an der Havel, Germany [↑](#footnote-ref-2)
